# A Bayesian Approach to the Evolution of Metabolic Networks on a Phylogeny

**DOI:** 10.1371/journal.pcbi.1000868

**Published:** 2010-08-05

**Authors:** Aziz Mithani, Gail M. Preston, Jotun Hein

**Affiliations:** 1 Department of Statistics, University of Oxford, Oxford, United Kingdom; 2 Department of Plant Sciences, University of Oxford, Oxford, United Kingdom; Broad Institute of MIT and Harvard, United States of America

## Abstract

The availability of genomes of many closely related bacteria with diverse metabolic capabilities offers the possibility of tracing metabolic evolution on a phylogeny relating the genomes to understand the evolutionary processes and constraints that affect the evolution of metabolic networks. Using simple (independent loss/gain of reactions) or complex (incorporating dependencies among reactions) stochastic models of metabolic evolution, it is possible to study how metabolic networks evolve over time. Here, we describe a model that takes the reaction neighborhood into account when modeling metabolic evolution. The model also allows estimation of the strength of the neighborhood effect during the course of evolution. We present Gibbs samplers for sampling networks at the internal node of a phylogeny and for estimating the parameters of evolution over a phylogeny without exploring the whole search space by iteratively sampling from the conditional distributions of the internal networks and parameters. The samplers are used to estimate the parameters of evolution of metabolic networks of bacteria in the genus *Pseudomonas* and to infer the metabolic networks of the ancestral pseudomonads. The results suggest that pathway maps that are conserved across the *Pseudomonas* phylogeny have a stronger neighborhood structure than those which have a variable distribution of reactions across the phylogeny, and that some *Pseudomonas* lineages are going through genome reduction resulting in the loss of a number of reactions from their metabolic networks.

## Author Summary

Metabolic networks correspond to one of the most complex cellular processes. Most organisms have a common set of reactions as a part of their metabolic networks that relate to essential processes such as generation of energy and the synthesis of important biological molecules, which are required for their survival. However, a large proportion of the reactions present in different organisms are specific to the needs of individual organisms. The regions of metabolic networks corresponding to these non-essential reactions are under continuous evolution. Using different models of evolution, we can ask important biological questions about the ways in which the metabolic networks of different organisms enable them to be well-adapted to the environments in which they live, and how these metabolic adaptations have evolved. We use a stochastic approach to study the evolution of metabolic networks and show that evolutionary inferences can be made using the structure of these networks. Our results indicate that plant pathogenic *Pseudomonas* are going through genome reduction resulting in the loss of metabolic functionalities. We also show the potential of stochastic approaches to infer the networks present at ancestral levels of a given phylogeny compared to deterministic methods such as parsimony.

## Introduction

Biological networks are under continuous evolution and their evolution is one of the major areas of research today [1]–[6]. The evolution of biological networks can be studied using various approaches such as maximum likelihood and parsimony [7], [8]. The maximum likelihood approach calculates the likelihood of evolution of one network into another by summing over all possible networks that can occur during the course of evolution under the given model. Parsimony, on the other hand, assumes minimum evolution and only considers those networks that correspond to the minimum number of changes between the two networks. However, the problem with these approaches is that enumeration of networks potentially occurring during evolution becomes impractical in the case of biological networks as the number of networks grows exponentially with the network size. Recently, the evolution of biological networks has been studied using stochastic approaches where efficient sampling techniques makes the problem computationally tractable. For example, Wiuf *et al.* [5] used importance sampling to approximate the likelihood and estimate parameters for the growth of protein networks under a duplicate attachment model. Similarly, Ratmann *et al.* [6] used approximate Bayesian computation to summarize key features of protein networks. The authors also approximated the posterior distribution of the model parameters for network growth using a Markov Chain Monte Carlo algorithm.

In this work, we focus on metabolic networks. The evolution of metabolic networks is characterized by gain and loss of reactions (or enzymes) connecting two or more metabolites and can be described as a discrete space continuous time Markov process where at each step of the network evolution a reaction is either added or deleted until the desired network is obtained [9]. To give a biologically relevant picture of evolution some reactions may be defined as core (reactions that cannot be deleted during the course of evolution) or prohibited (reactions that cannot be added) in the given networks. The evolution of metabolic networks can then be studied using simple (independent loss/gain of reactions) or complex (incorporating dependencies among reactions) stochastic models of metabolic evolution. We previously presented a neighbor-dependent model for the insertion and deletion of edges from a network where the rates with which reactions are added or removed from a network depend on the fraction of neighboring reactions present in the network [9]. In this model, two reactions were considered to be neighbors if they shared at least one metabolite. The model is summarized in Section ‘Neighbor-dependent model’ below. The neighbor-dependent model depicts a biologically relevant picture of metabolic evolution by taking the network structure into account when calculating the rates of insertion and deletion of reactions from a network. The model is, however, limited in the sense that it does not allow one to measure the strength of the neighborhood structure affecting network evolution.

Here, we present an extended model called the hybrid model that combines an independent edge model, where edges are gained or lost independently, and a neighbor-dependent model of network evolution [9] such that the rate of going from one network to another is a sum of the rates under the two models based on a parameter, which measures the probability of being in the neighbor dependent model. This allows estimation of the neighborhood effect during metabolic evolution. When modeling network evolution, we represent metabolic networks as directed hypergraphs [9]–[11], where an edge called a hyperedge represents a reaction and may connect any number of vertices or metabolites. Representing metabolic networks as hypergraphs not only captures the relationship between multiple metabolites involved in a reaction but also provides an intuitive approach to study evolution since loss or gain of reactions can be regarded as loss or gain of hyperedges.

We use the hybrid model to study the evolution of a set of metabolic networks connected over a phylogeny. Previous attempts to study the evolution of metabolic networks in a phylogenetic context include Dandekar *et al.* [12] and Peregrin *et al.* [13]. However, to our knowledge, the stochastic treatment of metabolic evolution over a phylogeny is an unexplored area. Here, the phylogenetic relationship between the networks is established using sequence data since the metabolic annotations available for the majority of genome-sequenced organisms are generated using automated annotation tools based on the similarity of predicted genes to genes of known function and, therefore, contain a huge amount of noise. In addition, we treat the branch lengths obtained using the sequence data as certain. The advantage of fixing branch lengths is that the calculations do not require summing over all branch lengths for the given tree. Calculating the likelihood over a phylogeny then requires a sum, over all possible networks that may have existed at the interior nodes of the tree, of the probabilities of each scenario of events. This is similar to the idea introduced by Felsenstein [14] for observing DNA sequences over a phylogeny. To sample the networks at internal nodes of the tree a Gibbs sampler [15], [16] is presented that samples a network conditioned on its three neighbors, including a parent and two children networks, for given parameter values. A Gibbs sampler for estimating the parameters of evolution that encases the Gibbs sampler for internal networks sampling is also presented. The sampler estimates the evolution parameters without exploring the whole search space by iteratively sampling from the conditional distributions of the trees and parameters. We demonstrate the Gibbs sampler by estimating and comparing the evolution parameters for the metabolic networks of bacteria belonging to the genus *Pseudomonas*. The Gibbs sampler can also be used to infer the ancestral networks of a given phylogeny. This is shown by inferring the metabolic networks of *Pseudomonas* spp. ancestors.

## Methods

### Neighbor-dependent model

In the neighbor-dependent for the evolution of metabolic networks [9] hyperedges are inserted or deleted from a network depending on the fraction of neighboring hyperedges present in the network. Two hyperedges are considered as neighbors if they share a node. The model assumes that the number of nodes in a network remains fixed and there is a set 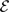 such that 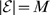 of hyperedges connecting these nodes. The model also assumes the existence of a network called *Reference Network* which contains all these hyperedges. If the hyperedges in the reference network are labeled 1 to 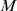 then any given network 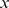 can be represented as a sequence of 0s and 1s such that the 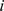-th entry 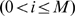 in the sequence is 1 if and only if the hyperedge labeled 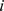 is present in the network 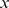, and 0 otherwise. Let the rate matrix describing the evolution under the neighbor-dependent model be denoted by 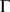. An entry 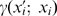 in this rate matrix corresponds to the rate of going from a network 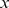 to a network 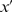, which differs from 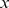 at position 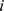. In the neighbor-dependent model, the rate 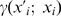 of going from 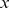 to 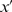 depends on 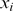, 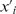 and the neighboring hyperedges 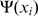 present in the network 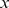, and is given as follows:
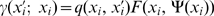
(1)where the function 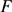 corresponds to the neighborhood component and 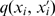 is the appropriate entry from the 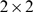 rate matrix 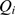 for the hyperedge 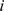. The rate matrix 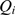 is given as
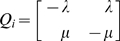
(2)where 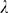 is the insertion rate and 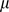 is the deletion rate.

The neighborhood component 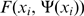 weights the insertion and deletion rates by the proportion of neighbors present in the network and is given as follows:
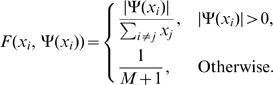
(3)The denominator 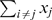 in Equation 3 gives the number of hyperedges present in the current network.

### Hybrid model of network evolution

Although the neighbor-dependent model summarized above produces a biologically relevant behavior whereby highly connected reactions are toggled more frequently than the poorly connected counterparts, it does not allow one to determine the strength of the neighborhood structure effecting the evolution of metabolic networks. To overcome this limitation, a parameter can be introduced in the model that corresponds to the neighborhood effect during the course of metabolic network evolution.

Consider two networks 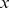 and 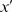 which differ at position 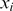. The hybrid model combines the independent edge model where edges are added or deleted independently, and the neighbor-dependent model summarized above such that the rate of going from 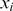 to 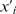 is the sum of the rates under the two models based on a parameter 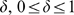, which specifies the probability of being in the neighbor-dependent model. The rate from 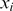 to 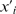 is given as
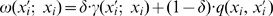
where the term 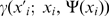 is the rate under the neighbor-dependent model given by Equation 1 and the term 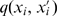 is the rate under the independent edge model corresponding to the appropriate entry from the rate matrix Q given by Equation 2. Substituting the value of 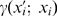 from Equation 1, the above equation can be simplified as follows.
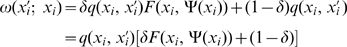
(4)where the term 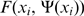 corresponds to the neighborhood component given by Equation 3.

**Figure 1:**
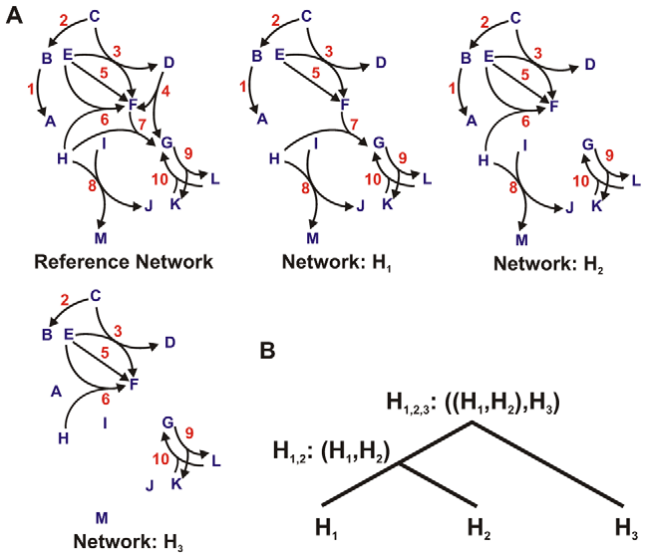
Toy networks connected by a phylogeny. (A) Toy networks consisting of 13 nodes. The nodes are labeled from A to M (blue) and the hyperedges are labeled from 1 to 10 (red). The reference network consists of all allowed hyperedges for this example system. Networks 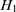, 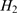 and 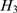 consist of subsets of the hyperedges from the reference network. (B) A phylogeny connecting the networks 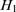, 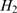 and 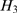.

It can been seen from (4) that the model behaves under the independent edge model when 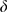 equals 0 and under the neighbor-dependent model described in the previous section when 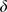 equals 1. For example, consider the toy network 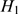 shown in Figure 1A. The reference network 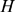 containing all allowed hyperedges for this example system is also shown in the figure. The system behavior for different values of 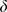 is illustrated in Figure S1 for the toy network 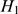 when simulated under the hybrid model along with the number of neighbors for each hyperedge. The rates were calculated at each step using (4). An edge was then selected based on these rates and was inserted if absent from the current network and deleted otherwise. As expected, hyperedges evolve independently when 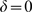, resulting in similar insertion frequencies for all hyperedges and increasingly reflecting their neighborhood as the value of 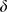 goes up to unity. The fitness of the model is discussed in the Section ‘Fitness of the hybrid model’ below.

### Evolution on a phylogeny

Biological networks are connected over a phylogenetic tree which is known through sequence analysis. Calculating the likelihood over a phylogeny requires a sum, over all possible networks that may have existed at the interior nodes of the tree, of the probabilities of each scenario of events. For example, Figure 1A shows an example system containing three networks 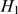, 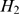 and 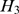 with a phylogeny connecting the three networks shown in Figure 1B. Let the phylogenetic tree be denoted by 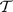. The likelihood of the tree 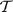 is given as follows.
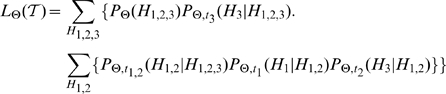
(5)


Here 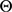 denotes the parameters of the model, which is 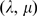 in the case of the neighbor-dependent model and 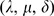 in the case of the hybrid model. 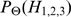 is the marginal probability of observing the root and 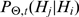 denotes the pairwise likelihood of evolving from the network 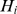 to the network 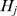 conditioned on 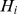 in time 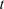 for the given parameters.

In general, the likelihood of a tree with more than three networks can be calculated using the recursion described by Felsenstein [17]. The likelihood at an internal node 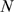 of the tree is given by the following recurrence relation
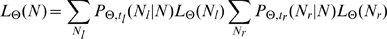
(6)where 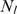 and 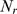 are left and right descendants of the node 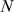. The likelihood of the complete tree 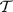 is then given as
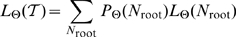
(7)where 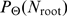 is the marginal probability of observing the root and 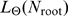 is given by Equation 6.

Evaluating Equations 5 and 7 requires an algorithm to systematically and efficiently sample networks at the internal nodes of a tree and a method to calculate the pairwise likelihood of network evolution. A Metropolis-Hastings algorithm to calculate the pairwise likelihood based on sampling paths between network pairs was described by Mithani *et al.* [9], which calculates the likelihood by summing over paths between the given network pairs. To sample networks at the internal node of a tree, a Markov chain can be constructed where states correspond to networks at the internal nodes. The networks can then be sampled using a Gibbs sampler [15], [16] as described in the next section.

### Sampling internal nodes

Given a set of networks related by a phylogenetic tree, the networks at the internal nodes of the tree can be sampled using a Gibbs sampler. The general idea is to sample each internal network by conditioning on its three neighbors (one parent and two children). This approach for sampling internal networks is similar to the one used by Holmes and Bruno [18] for DNA sequence alignment. However, instead of using linear sequences, the sampler takes into account the network structure when calculating the new state. The procedure is described below.

Consider a network 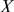 with its three neighbors 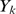 with branch lengths 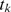, 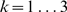. The new network 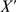 is selected as follows.

1. For each hyperedge 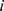, calculate the 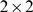 rate matrix
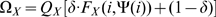
where 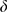 is the neighbor-dependence probability, 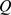 is the rate matrix given by Equation 2 and the function 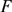 corresponds to the neighborhood component given by Equation 3.
2. Calculate, for each neighbor 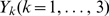, the transition probabilities 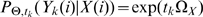.
3. Sample the new state 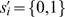 for hyperedge 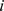 from the distribution
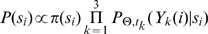
(8)where 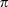 is the vector equilibrium probabilities and can be obtained by solving the equation 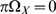.

**Figure 2:**
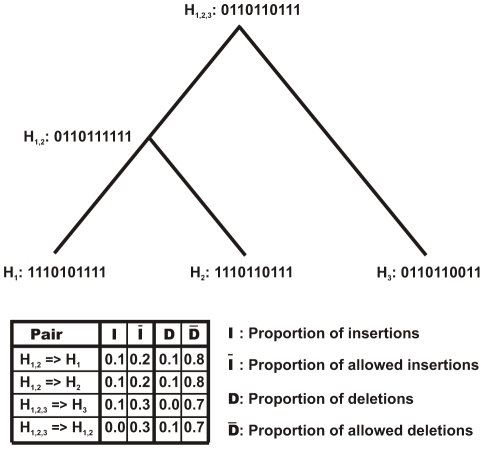
A sample phylogenetic tree for the toy networks shown in **Figure 1**. The tree contains arbitrary networks assigned at the internal nodes. Also shown are the proportion of insertion and deletion events and the proportion of allowed insertion and deletion events while going from various ancestral networks to descendant networks.

**Example** Consider the network 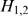 in Figure 2 for which new state is to be calculated. Denote the network by 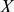. The three neighboring networks of the network 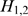 are the networks 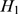, 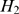 and 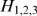 labeled as 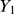, 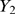 and 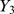 respectively. If 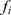 denotes the neighborhood component for hyperedge 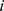 then for the given rate parameters 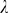 (insertion) and 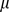 (deletion), and the neighbor-dependence probability 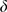 the rate matrix 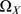 is written as
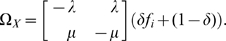
For simplicity, assume that 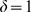. The system then behaves under the neighbor-dependent model and the rate matrix simplifies to
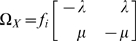
The transition probability matrix of transforming 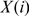 to 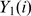 is then given as
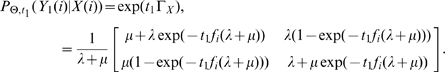
The transition probability matrices 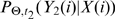 and 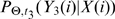 can be calculated in the similar fashion.

Once the transition probability matrices have been obtained, the sample for the new network 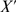 can be drawn using Equation 8. For example, if the current configuration of the networks are taken as shown in Figure 2, then the sample for the new state 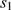, for hyperedge 1 is drawn from the following distribution:
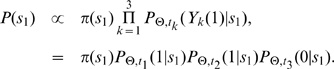



The samples for hyperedges labeled 2 to 10 can be drawn in a similar fashion to obtain the new network.

### Estimation of parameters

The Gibbs sampler described above samples the internal networks on a phylogenetic tree for given parameter values. This can be extended to estimate the parameters 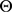 of evolution where 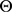 equals (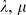) in case of the neighbor-dependent model and (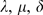) in case of the hybrid model. One way is to nest it within another Gibbs Sampler which iteratively samples internal networks and parameters from the distributions 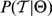 and 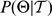 respectively. The general outline of the Gibbs sampler is as follows:

- Choose initial values for the parameters 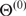.
- Generate 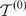 by using the procedure described in Section ‘Sampling internal nodes’ using 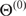.
- Use 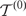 to generate 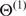 by drawing from the distribution 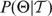.
- Repeat 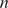 times to get subset of points 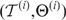, where 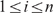, are the simulated estimates from the joint distribution 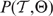.

The samples for parameters can be drawn using a Metropolis-Hastings algorithm [19], [20] as described next. Since the Metropolis-Hastings algorithm is a well-established method, it suffices here to give details about how a proposal for new parameters can be generated. Readers interested in the general details of the algorithm are referred to Chapter 1 of Gilks *et al.* [21]. The performance of the Gibbs sampler is discussed in Text S1.

### Parameter proposal

#### Rates proposal

For a given tree 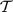, a proposal for the rate parameters can be generated from a gamma distribution
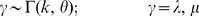
where 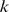 is the shape parameter and 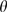 is the scale parameter. The hyper-parameters 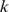 and 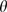 can be calculated from the given tree as described next.

Starting from root, calculate the proportion of insertion events 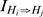 and the proportion of deletion events 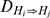 between the parent network 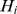 and the child network 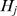 in the given tree 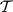 by dividing the number of insertion and deletion events by the total number of alterable hyperedges 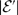 in the system. Also, calculate the proportion of allowed insertion and deletion events between these pairs. Let these be denoted by 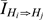 and 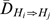. The hyper-parameters 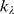 and 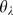 for sampling insertion rate can then be given as
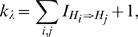
(9)

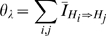
(10)Similarly, the hyper-parameters 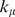 and 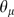 for sampling deletion rate are given as
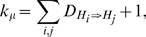
(11)

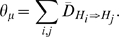
(12)


#### Example

The calculation of hyper-parameters 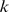 and 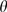 is demonstrated on the tree shown in Figure 2 connecting the toy networks shown in Figure 1. The number of hyperedges in the reference network is 10. If no core or prohibited hyperedges are assumed, then the number of alterable hyperedges 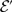 is also 10, i.e. 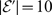. Going from the network 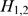 to the network 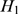 there is one insertion event and one deletion event out of 2 and 8 allowed insertion and deletion events respectively resulting in the following values:
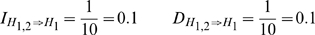


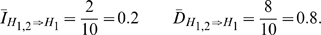
The same is true for going from the network pair 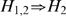. Values for other network pairs can be calculated in a similar fashion. The values for 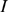, 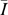, 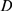 and 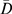 for all parent-child pairs in the example tree are listed in Figure 2. Using Equations 9 and 10, the hyper-parameters for sampling the insertion rate are calculated as
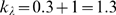


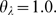
Similarly, using Equations 11 and 12 the hyper-parameters for sampling the deletion rate become
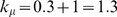


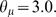


#### Dependence probability proposal

The hybrid model for metabolic network evolution described above allows estimation of the neighborhood effect shaping the evolution of given set of networks. The proposal for the parameter 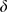 measuring the probability of being in the neighbor-dependent model can be generated from a beta distribution
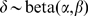
where the hyper-parameters 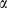 and 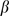 are the shape parameters and are calculated as follows.

Calculate the average number of neighbors present in the networks present at the leaves of the phylogeny. For example, if the network 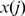 is a leaf network, i.e. it occurs at the tip of the given phylogenetic tree, then calculate
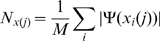
The parameter 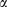 is then given as the mean of the average number of neighbors present in all the networks present at the leaves of the given phylogenetic tree. For a tree 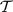 with 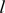 leaves, this can be written as follows.
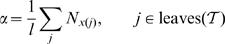
(13)


The shape parameter 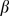 corresponds to the average number of neighbors in the reference network (REF) and is given as
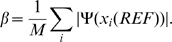
(14)


### Proposal probability

#### Rates proposal

The proposal probability 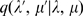 for the rate parameters is given as
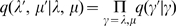
such that
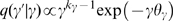
where 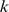 and 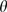 are the hyper-parameters of the gamma distribution given by Equations 9 and 10 respectively when 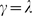 and by Equations 11 and 12 otherwise.

#### Dependence probability proposal

The proposal probability 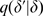 for the dependence probability parameters is given as
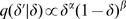
where 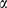 and 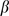 are the shape parameters of the beta distribution calculated using Equations 13 and 14 respectively.

### Pairwise likelihood

The Metropolis-Hastings procedure described above to sample parameters requires the likelihood of the tree when moving in the parameter space. The likelihood can be calculated using Equation 5 which in turn requires calculation of the pairwise likelihood between network pairs. The pairwise likelihood can be calculated using the Metropolis-Hastings algorithm described in Mithani *et al.* [9] which calculates the likelihood by summing over all paths between the given network pair. However, for the Gibbs sampler described above in Section ‘Estimation of parameters’ this seems impractical since it will require running the Metropolis-Hastings sampler for all network pairs. An alternate way is to use a pseudo-likelihood value when calculating the acceptance probability for parameters. We calculate the pseudo-likelihood for a given network pair by dividing the network into smaller sub-networks and multiplying the pairwise likelihoods of the individual sub-networks.

Let 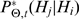 denote the pseudo-likelihood from the network 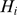 to the network 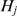 in time 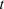 for the given parameter values. This is given as
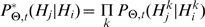
where 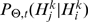 is the pairwise likelihood of evolving sub-network 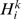 into 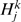 calculated by solving the exponential 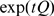. The procedure to obtain sub-networks 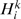 containing at most 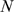 hyperedges is outlined below.

1. Initialize 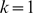.
2. Select the hyperedge 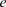 with highest number of neighbors and add it to the network 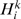.
3. Add the top 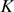 neighbors of hyperedge 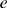 based on the number of neighbors to the network 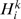 where 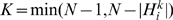.
4. Remove the hyperedges present in 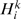 from 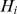 and calculate the number of neighbors based on the remaining hyperedges in the network.
5. Repeat steps 2–4 until 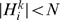.
6. Increment 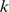 and repeat steps 2–5 until the network 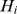 is exhausted.

**Figure 3:**
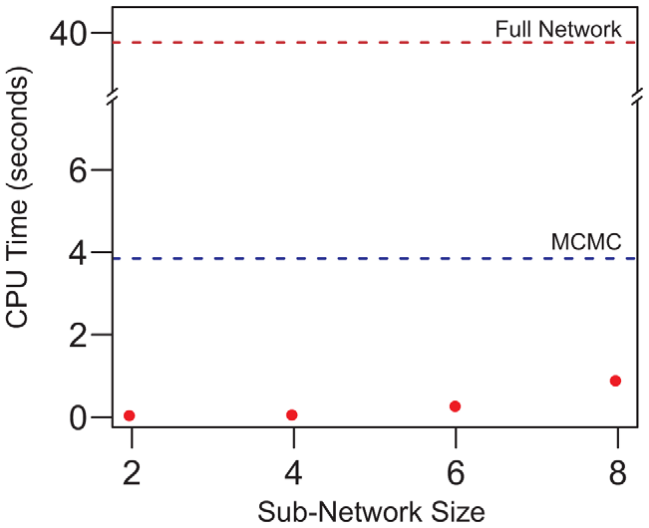
Average CPU time taken for calculating the pseudo-likelihood on toy networks. The pseudo-likelihood of going from the network 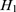 to the network 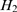 (Figure 1) was calculated conditioned on 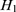 for different sub-network sizes. The times taken for calculating the pseudo-likelihood averaged across three runs are shown in red. The horizontal lines show the average CPU time taken using the MCMC approach based on path sampling described in Mithani *et al.* [9] with 11,000 iterations including first 1,000 iteration as burn-in period (blue dashed line) and by exponentiating the full network (brown dotted line).

An example is given in Figure S2, which shows the sub-networks for the toy network 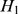 shown in Figure 1 for different values of 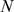. The above procedure was used to calculate the pseudo-likelihood of evolution of the toy network 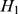 to the network 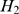 (Figure 1A) for different subnetwork sizes, and the results were compared against the likelihood obtained by the MCMC approach described in Mithani *et al.* [9] and the true likelihood values obtained by evaluating 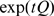. All likelihood values were conditioned on the starting network. The average CPU time taken by different approaches is shown in Figure 3 and the pseudo-likelihood values are listed in Table S1. The sub-network approach provides a reasonable approximation of the likelihood with a significant time advantage over the MCMC approach.

## Results

### Fitness of the hybrid model

To see if the hybrid model fitted the metabolic network data better than the neighbor-dependent model, a likelihood ratio test was performed using the metabolic data for the bacteria belonging to the genus *Pseudomonas*. The results show that the hybrid model fits the metabolic data better than the neighbor-dependent model. For example, consider the metabolic networks in *Pseudomonas fluorescens* Pf0-1. The maximum likelihood estimates (MLEs) for the evolution of glycolysis/gluconeogenesis map [22] from *Pseudomonas fluorescens* Pf-5 to *P. fluorescens* Pf0-1 obtained using the Gibbs sampler described by Mithani *et al.* [9] were 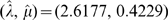 under the neighbor-dependent model and 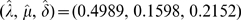 under the hybrid model. Using the MLEs, the likelihood of observing the data under each model was calculated. Assuming that evolution has been taking place for a long time, it is reasonable to use the equilibrium probability of a network to approximate the probability of observing the network. The equilibrium probabilities were calculated using the procedure described by Mithani *et al.* [9]. The maximum log likelihood obtained under the neighbor-dependent model equaled −76.53 whereas the maximum log likelihood obtained under the hybrid model equaled −63.47. The likelihood ratio test statistic 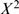 was calculated as 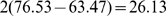 under 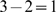 degree of freedom. The 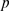-value 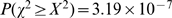 on 1 degree of freedom suggests that the hybrid model fits the data better than the neighbor-dependent model. The MLEs, maximum log-likelihoods and the 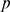-values for different pathway maps in *P. fluorescens* Pf0-1 used in this analysis are listed in Table 1. The low 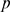-values for all the pathway maps suggest a better fit for the hybrid model compared to the neighbor-dependent model. Likelihood ratio tests for other genome-sequenced *Pseudomonas* strains used in this analysiss showed similar results (data not shown).

**Table 1:** Likelihood ratio test between the neighbor-dependent and hybrid models of metabolic evolution.

Pathway map	Neighbor-dependent model		Hybrid model		LH ratio	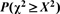
	( 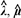 )	Log LH	( 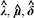 )	Log LH		
Glycolysis/Gluconeogenesis	(2.6177, 0.4229)	−76.53	(0.4989, 0.1598, 0.2152)	−63.47	26.13	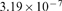
Pentose phosphate pathway	(0.5680, 0.7144)	−60.13	(0.4762, 0.2953, 0.4259)	−53.42	13.41	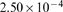
Lysine degradation	(0.0127, 1.0780)	−59.43	(0.0063, 0.2926, 0.0159)	−52.40	14.05	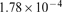
Histidine metabolism	(0.7669, 0.3895)	−54.22	(0.1852, 0.1643, 0.1370)	−47.28	13.89	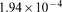
Phenylalanine metabolism	(1.1035, 0.6856)	−62.40	(1.0299, 1.0297, 0.0038)	−49.91	24.97	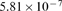
Pyruvate metabolism	(0.1648, 0.5656)	−88.64	(0.0897, 0.1913, 0.1194)	−81.74	13.81	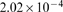

The maximum likelihood estimates (MLEs) of the parameter values (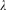: insertion rate, 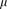: deletion rate and 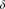: neighbor dependence probability), maximum log-likelihoods, likelihood (LH) ratios, and the 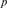-values for different pathway maps in *P. fluorescens* Pf0-1 used in this analysis. The MLEs were obtained using the Gibbs sampler described by Mithani *et al.* [9] by evolving the networks from *P. fluorescens* Pf-5 to *P. fluorescens* Pf0-1. The equilibrium probability of a network was used as the likelihood of observing the network. The low 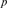-values for all the pathway maps suggest a better fit for the hybrid model compared to the neighbor-dependent model.

**Figure 4:**
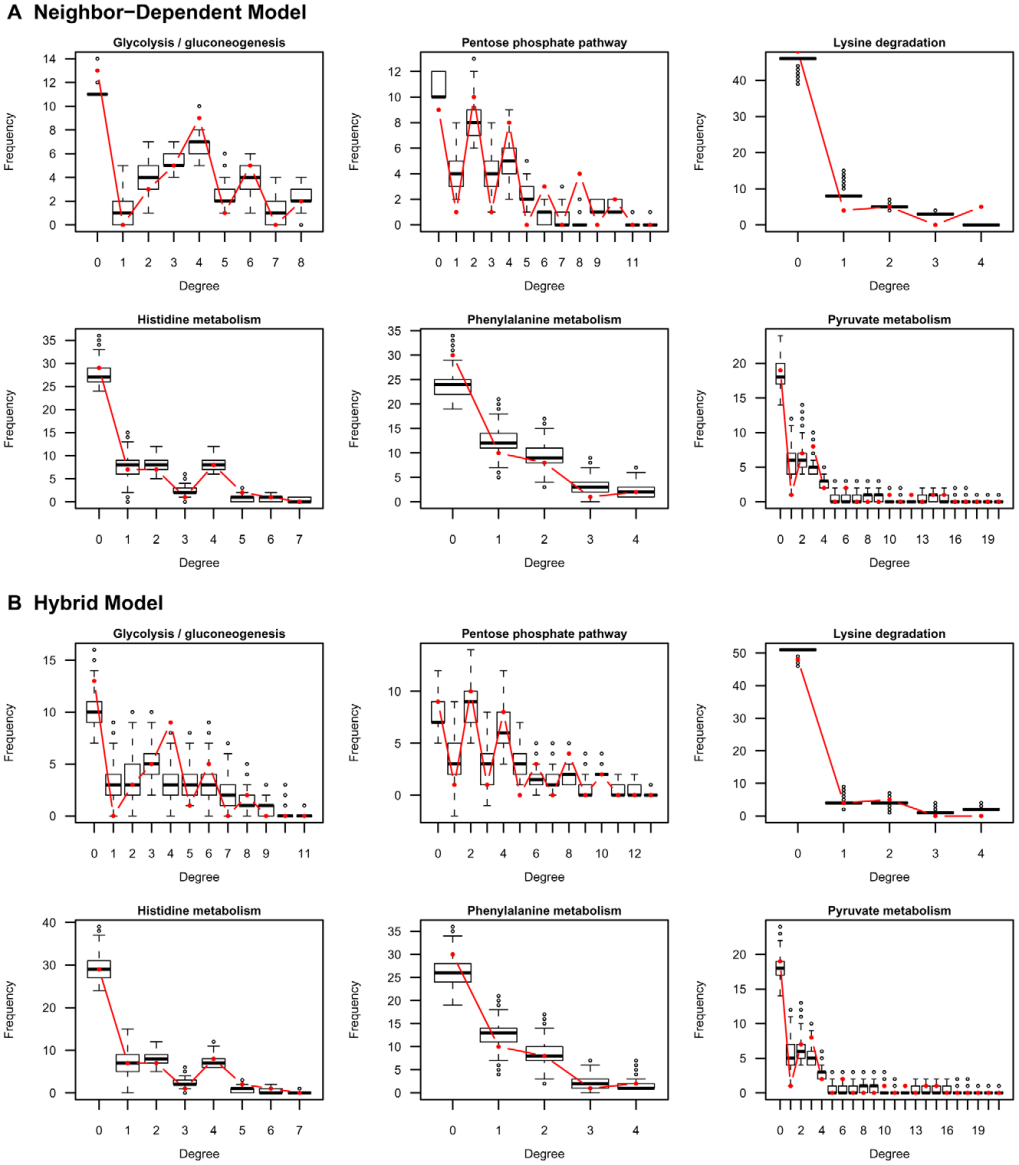
Degree distributions of nodes under the neighbor-dependent and hybrid models. Boxplots showing the degree distributions of nodes obtained by simulating the evolution for the pentose phosphate pathway, lysine degradation and phenylalanine metabolism maps in *P. fluorescens* Pf0-1 under (A) the neighbor-dependent model and (B) the hybrid model. The red line plots the actual degree distributions observed in the corresponding pathway map in *P. fluorescens* Pf0-1.

The fit of the data was further tested by comparing the degree distributions of the nodes obtained by simulating network evolution under the neighbor-dependent and hybrid models. The MLEs for the evolution of networks obtained under the two models were used as the simulation parameters. For example, when evolving the pathway maps in *P. fluorescens* Pf0-1, the parameter values listed in Table 1 were used. A total of 60,000 iterations were run with the first 10,000 iteration regarded as burn-in period. Samples were collected every 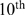 iteration and degree distributions were calculated. The results for the six pathway maps used in this analysis are shown in Figure 4 for *P. fluorescens* Pf0-1 as an example which suggest a better fit for the hybrid model than the neighbor-dependent model. Similar results (data not shown) were obtained for the other genome sequenced *Pseudomonas* strains used in this analysis.

### Toy networks

To test the Gibbs sampler described in Section ‘Sampling internal nodes’, the three network phylogeny shown in Figure 1 was used. The networks were sampled at the internal nodes for different rate combinations with the neighbor-dependence probability 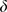 kept constant at 1. The likelihood value was then calculated using Equation 5 by summing over the networks visited by the sampler at each internal node for each rate combination. When calculating the likelihood over the phylogeny, the pairwise likelihood was calculated using matrix exponentiation. A total of 25,000 iterations were run for each rate combination with the first 10,000 iterations regarded as burn-in period. The exact likelihood of the phylogeny was also calculated by matrix exponentiation using all 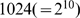 networks at each internal node. The likelihood values estimated using the networks visited by the Gibbs sampler were comparable to those obtained by summing over all 1024 networks. The true and estimated likelihood surfaces for a range of parameter values are shown in Figure S3.

**Figure 5:**
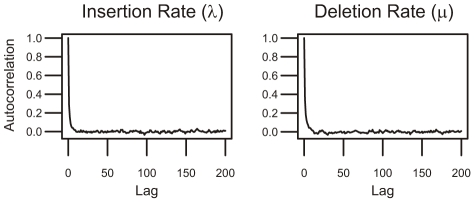
Autocorrelation of rate parameters. Parameters were estimated for the toy network phylogeny shown in Figure 1 using the Gibbs sampler described in Section ‘Estimation of parameters’. The values are averaged across three runs containing 60,000 iterations each.

We also ran the Gibbs sampler for parameter estimation for the toy networks. The sampler was run from a random starting value for 60,000 iterations with the first 10,000 iterations regarded as burn-in period. The samples were collected every 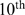 iteration to reduce computational overhead relating to storage as well as the correlation between samples. A sample MCMC trace for the first 1,000 iterations of the sampler for the rate parameters is shown in Figure S4. The autocorrelation of parameters is plotted in Figure 5 suggesting an exponential decrease in the correlation as the lag between the samples increases. To test the performance of the sampler, the likelihood of evolution for different rate combinations visited by the sampler was also calculated using Equation 5 by summing over networks visited by the sampler with 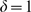. As before, the pairwise likelihood was evaluated by calculating the exponential of the rate matrix. The maximum likelihood averaged over three runs was found to be 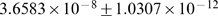 for parameters 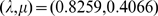 which is very close to the true likelihood obtained by matrix exponentiation (Figure S3).

### Parameter estimation for metabolic networks in *Pseudomonas*

**Figure 6:**
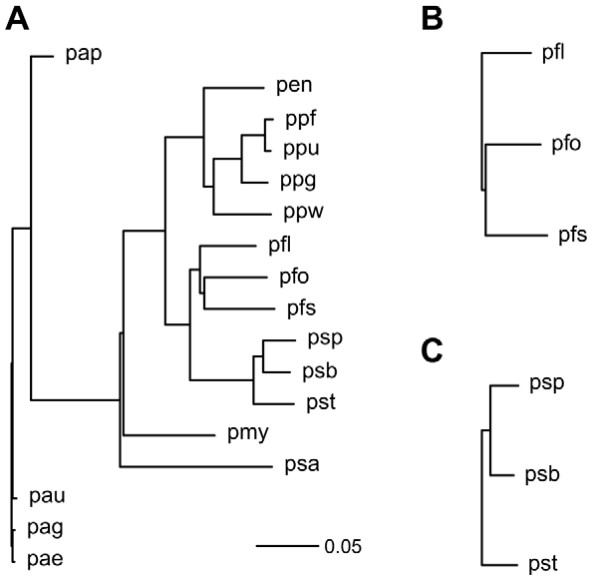
Phylogenies connecting bacteria belonging to the genus *Pseudomonas*. The phylogenies were generated using multi-locus sequence analysis of conserved housekeeping genes (*gapA*, *gltA*, *rpoD* and *gyrB*). (A) Phylogeny relating the seventeen genome-sequenced *Pseudomonas* strains. (B) Phylogeny relating the three strains of non pathogenic *P. fluorescens*. (C) Phylogeny relating the three strains of plant pathogenic *P. syringae*. Strain abbreviations: pae: *P. aeruginosa* PAO1, pap: *P. aeruginosa* PA7, pau: *P. aeruginosa* PA14, pag: *P. aeruginosa* LESB58, pen: *P. entomophila* L48, pfl: *P. fluorescens* Pf-5, pfo: *P. fluorescens* Pf0-1, pfs: *P. fluorescens* SBW25, pmy: *P. mendocina* ymp, ppf: *P. putida* F1, ppg: *P. putida* GB-1 ppu: *P. putida* KT2440, ppw: *P. putida* W619, psa: *P. stutzeri* A1501, psb: *P. syringae* pv. syringae B728a, psp: *P. syringae* pv. phaseolicola 1448A, and pst: *P. syringae* pv. tomato DC3000.

To study the metabolic evolution in bacteria, we used the Gibbs sampler to estimate the evolution parameters for the metabolic networks of bacteria belonging to the genus *Pseudomonas*. The diversity of pseudomonads, and the availability of genome-sequence data for multiple plant-associated *Pseudomonas fluorescens*, *Pseudomonas mendocina*, *Pseudomonas putida*, *Pseudomonas stutzeri* and *Pseudomonas syringae* strains, along with genome data for clinical isolates of *Pseudomonas aeruginosa* and for the insect pathogen *Pseudomonas entomophila* provide an excellent opportunity to use comparative genomic approaches to develop insight into the evolution of metabolic networks. The phylogeny connecting the seventeen genome-sequenced strains of *Pseudomonas* is shown in Figure 6A. The phylogeny was generated using multilocus sequencing analysis of conserved housekeeping genes ( *gltA*, *gapA*, *rpoD*, *gyrB*) [23]. The metabolic network data was extracted from the KEGG database [22] on 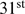 January 2010 for pathway maps across the seventeen *Pseudomonas* strains shown in Figure 6A using the Rahnuma tool [24]. The evolution parameters were also compared between two *Pseudomonas* species: *P. fluorescens*, a saprotroph that colonizes the soil environment, and *P. syringae*, a plant-pathogen that is found on leaf surfaces and in plant tissues. The phylogenetic relationships between these species is shown in Figures 6B and C. The results are discussed here for the six pathway maps listed in Table 2 as they provide a representative set of different neighborhood characteristics observed across the *Pseudomonas* strains used in this analysis. The basic information for each network across the seventeen *Pseudomonas* strains is given in Table S2.

**Table 2:** Posterior expectation and variance of evolution parameters estimated using the Gibbs sampler under the hybrid model.

Pathway Map	Phylogeny	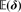	var( 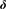 )	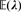	var( 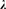 )	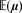	var( 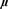 )	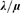
Glycolysis/Gluconeogenesis	((pfs,pfo),pfl)	0.2276	0.0054	2.0552	1.0280	1.2228	0.2653	1.6807
(MAP00010)	(pst,(psb,psp))	0.2404	0.0070	1.8645	3.3775	0.7280	0.2891	2.5611
	17 pseudomonads	0.1506	0.0034	0.7610	0.0117	0.7329	0.0114	1.0383
Pentose phosphate pathway	((pfs,pfo),pfl)	0.2785	0.0057	2.1103	0.5582	1.7194	0.3227	1.2273
(MAP00030)	(pst,(psb,psp))	0.3251	0.0071	1.8490	1.6921	1.0172	0.3212	1.8178
	17 pseudomonads	0.1863	0.0042	0.6762	0.0029	0.7462	0.0126	0.9062
Lysine degradation	((pfs,pfo),pfl)	0.0802	0.0032	0.9662	0.2795	1.6567	1.5943	0.5832
(MAP00310)	(pst,(psb,psp))	0.0637	0.0025	0.6986	0.1030	2.7245	2.8663	0.2564
	17 pseudomonads	0.0473	0.0030	0.4706	0.3492	0.6443	0.7188	0.7304
Histidine metabolism	((pfs,pfo),pfl)	0.1833	0.0065	1.6829	1.2133	1.0507	0.3456	1.6017
(MAP00340)	(pst,(psb,psp))	0.1749	0.0064	1.5321	0.9479	1.0735	0.3082	1.4272
	17 pseudomonads	0.0986	0.0022	0.8685	0.0203	0.6795	0.0057	1.2781
Phenylalanine metabolism	((pfs,pfo),pfl)	0.0783	0.0029	1.1686	0.2072	1.8255	0.9345	0.6402
(MAP00360)	(pst,(psb,psp))	0.0678	0.0024	1.0573	0.1448	2.2334	1.1112	0.4734
	17 pseudomonads	0.0617	0.0017	0.6004	0.0061	1.0723	0.0682	0.5599
Pyruvate metabolism	((pfs,pfo),pfl)	0.1413	0.0018	1.6497	0.3362	1.7913	0.4424	0.9210
(MAP00620)	(pst,(psb,psp))	0.1559	0.0020	1.5542	0.5376	1.2840	0.2888	1.2105
	17 pseudomonads	0.1119	0.0007	0.7668	0.0099	0.6838	0.0142	1.1213

Posterior expectation and variance of parameter values (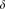: neighbor dependence probability, 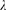: insertion rate and 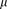: deletion rate) estimated using the Gibbs sampler under the hybrid model for the phylogenies relating the bacteria belonging to genus *Pseudomonas* (Figure 6). Hyperedges common to all seventeen genome-sequenced strains were defined as core and hyperedges missing in all seventeen strains were defined as prohibited hyperedges. The values are averaged over three runs of 60,000 iterations for *P. fluorescens* and *P. syringae* phylogenies, and 110,000 iterations for the phylogeny connecting the seventeen *Pseudomonas* strains with the first 10,000 iterations regarded as burn-in period in each case. Samples were collected every 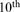 iteration. The codes MAPxxxxx correspond to the respective KEGG pathway codes [22]. Strain abbreviations: pfl: *P. fluorescens* Pf-5, pfo: *P. fluorescens* Pf0-1, pfs: *P. fluorescens* SBW25, psb: *P. syringae* pv. syringae B728a, psp: *P. syringae* pv. phaseolicola 1448A, and pst: *P. syringae* pv. tomato DC3000.

When estimating the parameters, the hyperedges corresponding to the reactions that were common to all seventeen *Pseudomonas* strains were defined as core edges and the hyperedges corresponding to the reactions not present in any of these seventeen species were defined as prohibited edges. Three independent replicates of the sampler were run from random starting values for 60,000 iterations for *P. fluorescens* and *P. syringae* phylogenies, and 110,000 iterations for the phylogeny connecting the seventeen *Pseudomonas* strains with the first 10,000 iterations regarded as burn-in period in each case. The samples were collected every 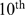 iteration to calculate the posterior expectations and variances of the parameters. These are listed in Table 2 and the ESS used for parameter estimation are listed in Table S3. The convergence of the algorithm was tested by checking the trace of the MCMC runs initiated from different starting values. An example is shown in Figure S5, which shows the trace for the sampler run on *P. fluorescens* phylogeny (Figure 6B). The running times and the acceptance percentages of the algorithm are listed in Table S4 for all three phylogenies. We also calculated the number of insertion and deletion events for each reaction as well as at each branch of the *Pseudomonas* phylogeny for all six pathway maps. These are shown in Figures S6 and S7.

The high insertion to deletion ratio (Table 2) for all three phylogenies for the glycolysis/gluconeogenesis map, pentose phosphate pathway map and pyruvate metabolism map, which are defined as a part of the carbohydrate metabolism of the bacteria in KEGG [22] and for the histidine metabolism map, which is a part of amino acid metabolism, suggests that very few reactions are missing from these networks in one or more *Pseudomonas* strains used in the analysis, resulting in a highly conserved network. Lysine and phenylalanine pathway maps, on the other hand, have higher deletion rates compared to the insertion rates suggesting a variable reaction distribution across the *Pseudomonas* phylogeny and instability of these functionalities. The results obtained in this study are consistent with the previous observation that the histidine metabolism map shows conservation of reactions across pseudomonads (Mithani, Hein and Preston, submitted) and that many *Pseudomonas* strains are able to use histidine as sole carbon and nitrogen source [25] whereas lysine and phenylalanine pathway maps have few conserved reactions across pseudomonads (Mithani, Hein and Preston, submitted) and are poor nutrient sources for these bacteria [25]. The results also indicate that the pathway maps which are highly conserved across the seventeen *Pseudomonas* strains, i.e. glycolysis/gluconeogenesis map, pentose phosphate pathway map, pyruvate metabolism map and histidine metabolism map, also have higher neighbor dependence probabilities compared to the other two pathway maps, which have variable reaction distribution across the *Pseudomonas* phylogeny. This might suggest a relationship between the neighborhood structure and the conservation of networks.

The comparison of the evolution parameters between *P. fluorescens* and *P. syringae* provides interesting insights into the evolution of the metabolic networks of these bacteria. For example, the insertion and deletion rates are generally higher in *P. fluorescens* than those in *P. syringae* suggesting a higher number of insertion and deletion events in *P. fluorescens* networks compared to *P. syringae* networks. This was expected since the evolutionary distance between the *P. fluorescens* strains is greater as compared to *P. syringae* strains (Figure 6) allowing more time for the networks in *P. fluorescens* to evolve. A higher deletion rate for lysine and phenylalanine pathway maps in *P. syringae* compared to *P. fluorescens*, however, suggests that *P. syringae* have had a higher number of deletion events than *P. fluorescens* during the course of evolution. This supports the finding that *P. syringae* have gone through a high number of deletion events than expected based on the comparison between observed and expected distribution of reactions across the *Pseudomonas* phylogeny, and the identification of reactions that are uniquely present or absent from a single lineage (Mithani, Hein and Preston, submitted). In addition, a very low insertion to deletion ratio (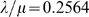) for lysine metabolism in *P. syringae* suggests a high number of deletion events in the lineage and consequently the loss of the ability of these bacteria to assimilate lysine. This is in agreement with nutrient utilization assays, which have reported that bacteria belonging to the species *P. syringae* do not assimilate lysine as a nutrient source [25]. Phenylalanine metabolism also has a higher deletion rate as compared to insertion rate in both *P. fluorescens* and *P. syringae* lineages. This in conjunction with experimental data reporting the weak ability of these bacteria to utilize phenylalanine as a nutrient source might lead to a hypothesis that both *P. fluorescens* and *P. syringae* are drifting towards losing their ability to assimilate phenylalanine. Overall, the results show that genome reduction is taking place in plant pathogenic bacteria belonging to the species *P. syringae* at a higher rate than their non-pathogenic counterparts in the species *P. fluorescens*.

### Ancestral network reconstruction

The final aim of this study was to infer reactions present in the common ancestor of *Pseudomonas* spp. and of individual species of *Pseudomonas*. One way to address this is to predict that the common ancestor contained all the reactions that are common to existing *Pseudomonas*. The variable reactions can then be assigned using a parsimonious approach which generates a conservative model of network evolution in which a minimum number of events occur. However, the results above suggest that some lineages, particularly *P. syringae*, have undergone deletion events relative to the common ancestor and that some reactions absent in one or more modern pseudomonads might be present in the ancestral strain. To take this into account, stochastic approaches such as the Gibbs sampler described in Section ‘Estimation of parameters’ can be used to sample ancestral networks from the posterior distribution of networks and the likelihood of reactions being present at various levels of the phylogeny can be calculated.

**Figure 7:**
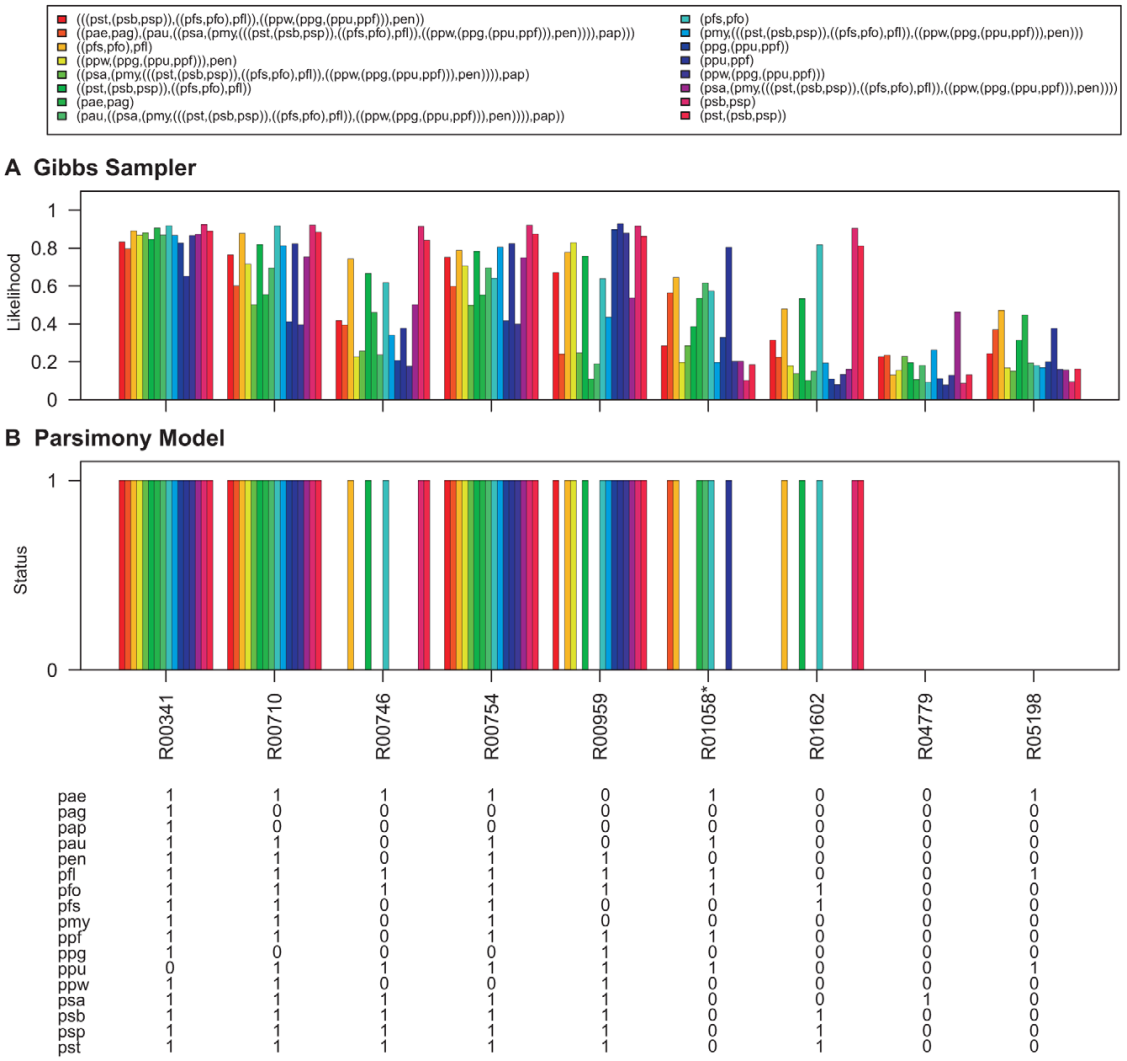
Ancestral network reconstruction for the glycolysis/gluconeogenesis map. The ancestral networks were reconstructed over the *Pseudomonas* phylogeny shown in Figure 6A. Also shown in the bottom panel is the distribution of reactions across different *Pseudomonas* strains. (A) Likelihood of being present for alterable reactions at various levels of *Pseudomonas* phylogeny obtained by calculating the proportion of times each hyperedge was present in the networks sampled by the Gibbs sampler. (B) Reaction status obtained under maximum parsimony calculated using the Fitch algorithm [26]. When assigning the reactions at the ancestral nodes the ties were resolved in favor of presence of reactions. Reactions for which parsimony failed to resolve ancestral predictions at the root are marked with an asterisk (*). Strain abbreviations: pae: *P. aeruginosa* PAO1, pap: *P. aeruginosa* PA7, pau: *P. aeruginosa* PA14, pag: *P. aeruginosa* LESB58, pen: *P. entomophila* L48, pfl: *P. fluorescens* Pf-5, pfo: *P. fluorescens* Pf0-1, pfs: *P. fluorescens* SBW25, pmy: *P. mendocina* ymp, ppf: *P. putida* F1, ppg: *P. putida* GB-1 ppu: *P. putida* KT2440, ppw: *P. putida* W619, psa: *P. stutzeri* A1501, psb: *P. syringae* pv. syringae B728a, psp: *P. syringae* pv. phaseolicola 1448A, and pst: *P. syringae* pv. tomato DC3000.

To demonstrate this, the Gibbs sampler was run on the pathway maps listed in Table 2. The Gibbs sampler was run with the same settings that were used for parameter estimation and samples for the networks at internal nodes of the *Pseudomonas* phylogeny (Figure 6A) were collected. The degree distributions of nodes at the ancestral levels of the phylogeny are given in Figures S8, S9, S10, S11, S12, S13. The likelihood of reactions being present at each level was obtained by calculating the proportion of times each hyperedge was present in the sampled networks. The results are shown in Figures 7A–[Fig pcbi-1000868-g008] [Fig pcbi-1000868-g009] [Fig pcbi-1000868-g010] [Fig pcbi-1000868-g011] 12A. Only alterable reactions, that is the reactions which were neither defined as core nor were defined as prohibited in the networks, are shown.

**Figure 8 pcbi-1000868-g008:**
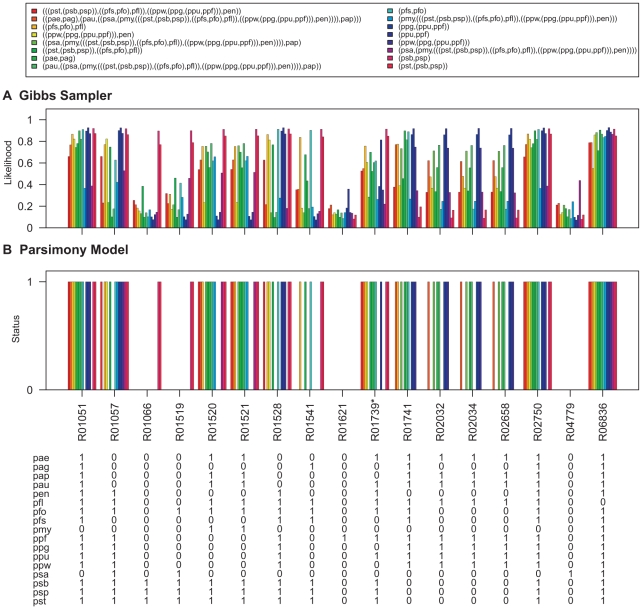
Ancestral network reconstruction for the pentose phosphate pathway map. The ancestral networks were reconstructed over the *Pseudomonas* phylogeny shown in Figure 6A. Also shown in the bottom panel is the distribution of reactions across different *Pseudomonas* strains. (A) Likelihood of being present for alterable reactions at various levels of *Pseudomonas* phylogeny obtained by calculating the proportion of times each hyperedge was present in the networks sampled by the Gibbs sampler. (B) Reaction status obtained under maximum parsimony calculated using the Fitch algorithm [26]. When assigning the reactions at the ancestral nodes the ties were resolved in favor of presence of reactions. Reactions for which parsimony failed to resolve ancestral predictions at the root are marked with an asterisk (*). Strain abbreviations: pae: *P. aeruginosa* PAO1, pap: *P. aeruginosa* PA7, pau: *P. aeruginosa* PA14, pag: *P. aeruginosa* LESB58, pen: *P. entomophila* L48, pfl: *P. fluorescens* Pf-5, pfo: *P. fluorescens* Pf0-1, pfs: *P. fluorescens* SBW25, pmy: *P. mendocina* ymp, ppf: *P. putida* F1, ppg: *P. putida* GB-1 ppu: *P. putida* KT2440, ppw: *P. putida* W619, psa: *P. stutzeri* A1501, psb: *P. syringae* pv. syringae B728a, psp: *P. syringae* pv. phaseolicola 1448A, and pst: *P. syringae* pv. tomato DC3000.

**Figure 9 pcbi-1000868-g009:**
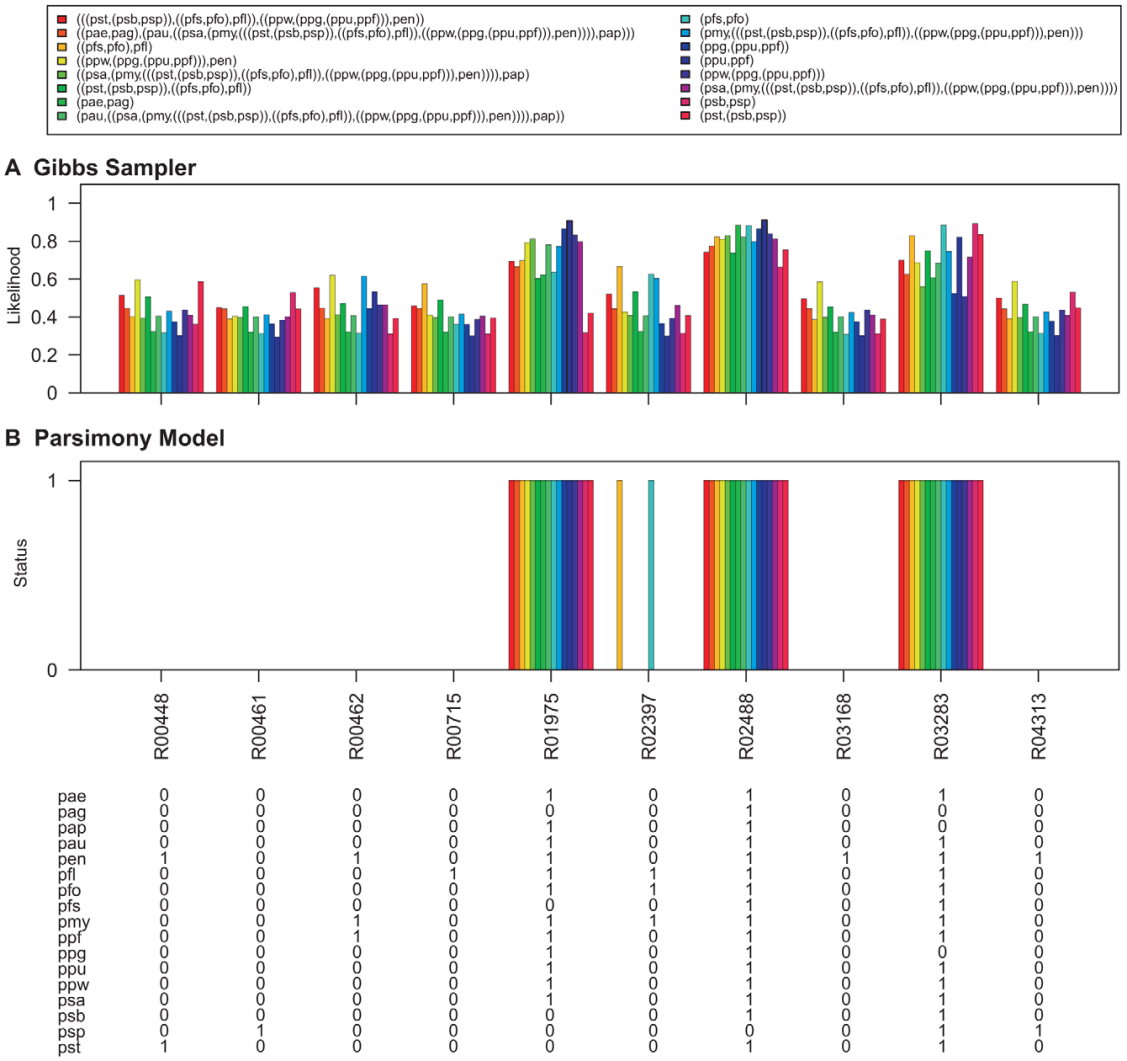
Ancestral network reconstruction for the lysine degradation map. The ancestral networks were reconstructed over the *Pseudomonas* phylogeny shown in Figure 6A. Also shown in the bottom panel is the distribution of reactions across different *Pseudomonas* strains. (A) Likelihood of being present for alterable reactions at various levels of *Pseudomonas* phylogeny obtained by calculating the proportion of times each hyperedge was present in the networks sampled by the Gibbs sampler. (B) Reaction status obtained under maximum parsimony calculated using the Fitch algorithm [26]. When assigning the reactions at the ancestral nodes the ties were resolved in favor of presence of reactions. Strain abbreviations: pae: *P. aeruginosa* PAO1, pap: *P. aeruginosa* PA7, pau: *P. aeruginosa* PA14, pag: *P. aeruginosa* LESB58, pen: *P. entomophila* L48, pfl: *P. fluorescens* Pf-5, pfo: *P. fluorescens* Pf0-1, pfs: *P. fluorescens* SBW25, pmy: *P. mendocina* ymp, ppf: *P. putida* F1, ppg: *P. putida* GB-1 ppu: *P. putida* KT2440, ppw: *P. putida* W619, psa: *P. stutzeri* A1501, psb: *P. syringae* pv. syringae B728a, psp: *P. syringae* pv. phaseolicola 1448A, and pst: *P. syringae* pv. tomato DC3000.

**Figure 10 pcbi-1000868-g010:**
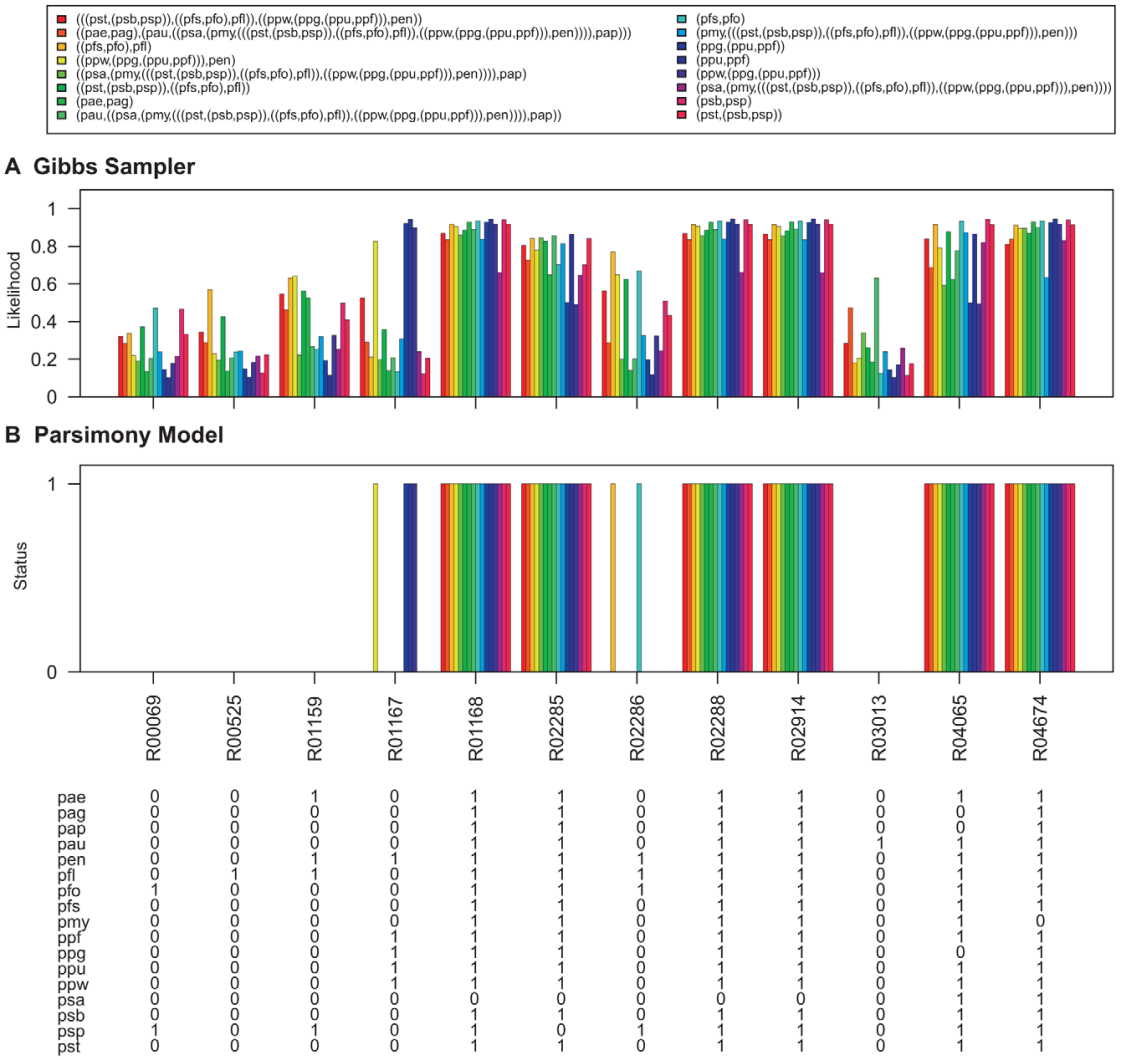
Ancestral network reconstruction for the histidine metabolism map. The ancestral networks were reconstructed over the *Pseudomonas* phylogeny shown in Figure 6A. Also shown in the bottom panel is the distribution of reactions across different *Pseudomonas* strains. (A) Likelihood of being present for alterable reactions at various levels of *Pseudomonas* phylogeny obtained by calculating the proportion of times each hyperedge was present in the networks sampled by the Gibbs sampler. (B) Reaction status obtained under maximum parsimony calculated using the Fitch algorithm [26]. When assigning the reactions at the ancestral nodes the ties were resolved in favor of presence of reactions. Strain abbreviations: pae: *P. aeruginosa* PAO1, pap: *P. aeruginosa* PA7, pau: *P. aeruginosa* PA14, pag: *P. aeruginosa* LESB58, pen: *P. entomophila* L48, pfl: *P. fluorescens* Pf-5, pfo: *P. fluorescens* Pf0-1, pfs: *P. fluorescens* SBW25, pmy: *P. mendocina* ymp, ppf: *P. putida* F1, ppg: *P. putida* GB-1 ppu: *P. putida* KT2440, ppw: *P. putida* W619, psa: *P. stutzeri* A1501, psb: *P. syringae* pv. syringae B728a, psp: *P. syringae* pv. phaseolicola 1448A, and pst: *P. syringae* pv. tomato DC3000.

**Figure 11 pcbi-1000868-g011:**
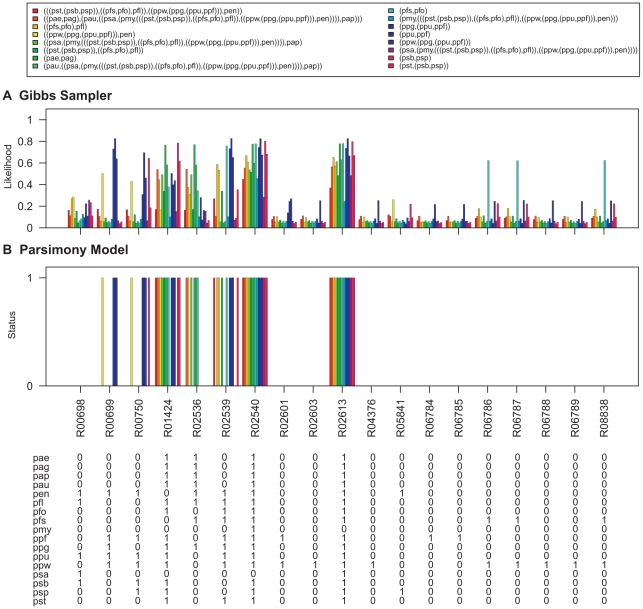
Ancestral network reconstruction for the phenylalanine metabolism map. The ancestral networks were reconstructed over the *Pseudomonas* phylogeny shown in Figure 6A. Also shown in the bottom panel is the distribution of reactions across different *Pseudomonas* strains. (A) Likelihood of being present for alterable reactions at various levels of *Pseudomonas* phylogeny obtained by calculating the proportion of times each hyperedge was present in the networks sampled by the Gibbs sampler. (B) Reaction status obtained under maximum parsimony calculated using the Fitch algorithm [26]. When assigning the reactions at the ancestral nodes the ties were resolved in favor of presence of reactions. Strain abbreviations: pae: *P. aeruginosa* PAO1, pap: *P. aeruginosa* PA7, pau: *P. aeruginosa* PA14, pag: *P. aeruginosa* LESB58, pen: *P. entomophila* L48, pfl: *P. fluorescens* Pf-5, pfo: *P. fluorescens* Pf0-1, pfs: *P. fluorescens* SBW25, pmy: *P. mendocina* ymp, ppf: *P. putida* F1, ppg: *P. putida* GB-1 ppu: *P. putida* KT2440, ppw: *P. putida* W619, psa: *P. stutzeri* A1501, psb: *P. syringae* pv. syringae B728a, psp: *P. syringae* pv. phaseolicola 1448A, and pst: *P. syringae* pv. tomato DC3000.

**Figure 12:**
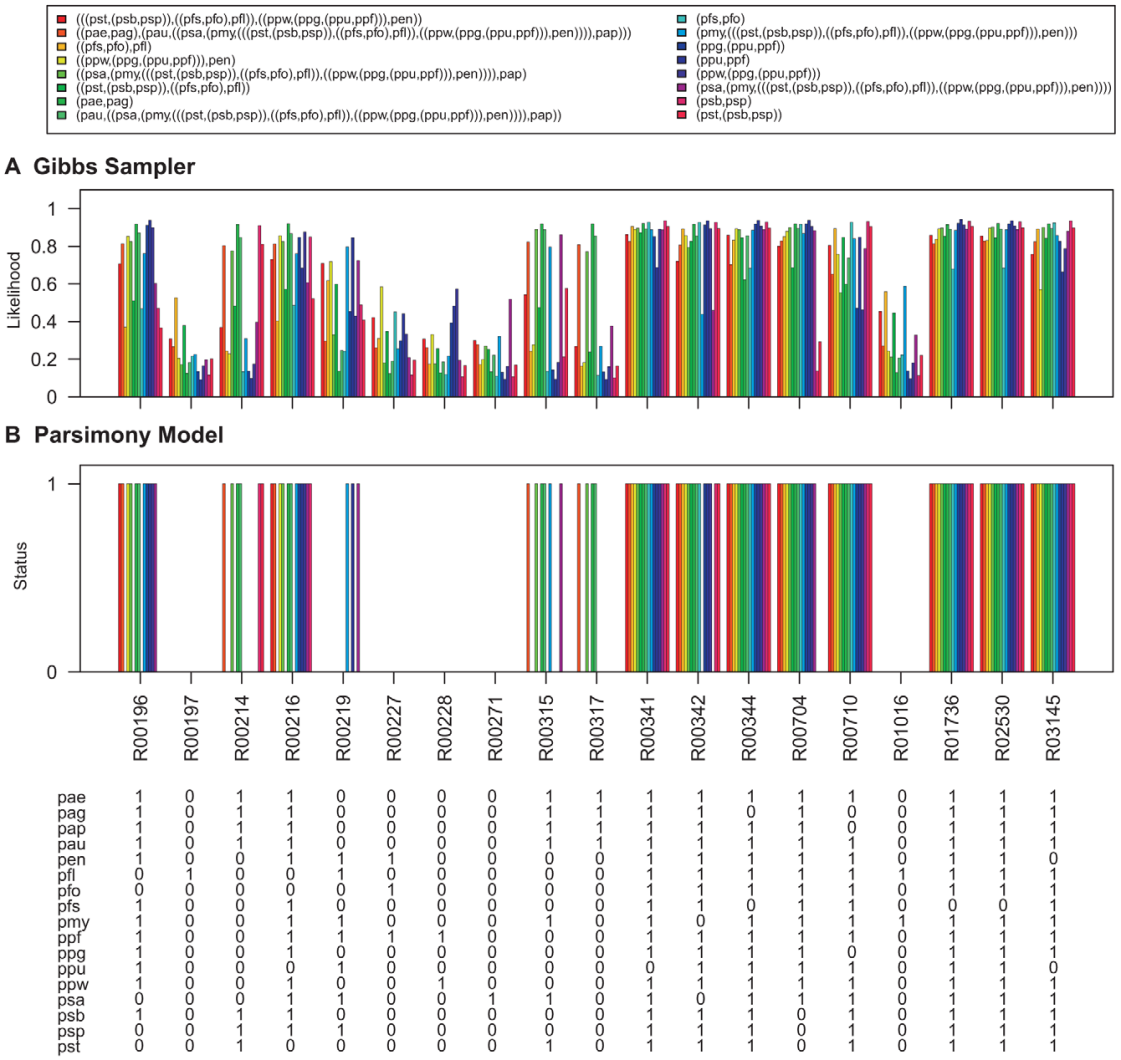
Ancestral network reconstruction for the pyruvate metabolism map. The ancestral networks were reconstructed over the *Pseudomonas* phylogeny shown in Figure 6A. Also shown in the bottom panel is the distribution of reactions across different *Pseudomonas* strains. (A) Likelihood of being present for alterable reactions at various levels of *Pseudomonas* phylogeny obtained by calculating the proportion of times each hyperedge was present in the networks sampled by the Gibbs sampler. (B) Reaction status obtained under maximum parsimony calculated using the Fitch algorithm [26]. When assigning the reactions at the ancestral nodes the ties were resolved in favor of presence of reactions. Strain abbreviations: pae: *P. aeruginosa* PAO1, pap: *P. aeruginosa* PA7, pau: *P. aeruginosa* PA14, pag: *P. aeruginosa* LESB58, pen: *P. entomophila* L48, pfl: *P. fluorescens* Pf-5, pfo: *P. fluorescens* Pf0-1, pfs: *P. fluorescens* SBW25, pmy: *P. mendocina* ymp, ppf: *P. putida* F1, ppg: *P. putida* GB-1 ppu: *P. putida* KT2440, ppw: *P. putida* W619, psa: *P. stutzeri* A1501, psb: *P. syringae* pv. syringae B728a, psp: *P. syringae* pv. phaseolicola 1448A, and pst: *P. syringae* pv. tomato DC3000.

The ancestral network reconstruction using the Gibbs sampler reported high likelihood values for reactions which are present in all the networks down a lineage and low likelihood values for reactions which show variable distributions across the *Pseudomonas* phylogeny. For example, in the pentose phosphate pathway map (Figure 8A), the reaction R01066, which is present only in the three *P. syringae* strains, was assigned a very high likelihood of being present in the common ancestor of *P. syringae* pv. phaseolicola 1448A and *P. syringae* pv. syringae B728a as well as in the common ancestor for all the tree *P. syringae* strains but a very low likelihood of being present for all other internal networks. In contrast, R06836, which is present in sixteen out of the seventeen *Pseudomonas* strains (absent in *P. fluorescens* Pf-5), is reported to have high likelihood values of being present in all internal networks of the phylogeny.

Ancestral predictions were also generated under the parsimony model for these networks using the Fitch Algorithm [26]. When assigning the reactions at the ancestral nodes the ties were resolved in favor of presence of reactions. The results are shown in Figures 7B–[Fig pcbi-1000868-g008] [Fig pcbi-1000868-g009] [Fig pcbi-1000868-g010] [Fig pcbi-1000868-g011] 12B. Reactions for which parsimony failed to resolve ancestral predictions at the root are marked with asterisks (*). Predictions generated for the *Pseudomonas* common ancestor using parsimony analysis are nearly identical to predictions generated for the *P. aeruginosa* common ancestor, which would be expected as parsimony assumes minimum evolution. In addition, parsimony generated a conservative model of network evolution in which a minimum number of events occur, but the stochastic approach takes network information into account when predicting ancestral networks. For example, in the case of the lysine degradation map (Figure 9), six out of the ten variable reactions are reported to be absent from all the ancestral networks of the *Pseudomonas* phylogeny using the parsimony approach whereas the stochastic approach taking the reaction neighborhood data into account assigns non-zero likelihood values to these reactions for being present in the ancestral pseudomonads. Similarly, all four reactions which are predicted to be absent from all ancestral pseudomonads in the histidine metabolism map under the parsimony model have non-zero likelihoods of being present in the ancestral networks using the stochastic approach (Figure 10). The results for ancestral network reconstruction for phenylalanine metabolism (Figure 11), on the other hand, suggested a very low level of conservation of reactions across the *Pseudomonas* phylogeny using both approaches and most of the variable reactions were predicted to be absent from the common ancestor. The variable distribution of these reactions across the seventeen *Pseudomonas* strains along with the results of ancestral network reconstruction suggests that these reactions might have been gained independently at organism level.

## Discussion

In this study, we have used a Bayesian approach to study the evolution of metabolic networks. We extended the neighbor-dependent model described by Mithani *et al.* [9] by introducing a parameter that estimates the probability of being present in the neighbor-dependent model. This not only provides a better fit for the data but also has an advantage over the existing model since it allows one to estimate the strength of neighborhood structure affecting the evolution of given networks. It must, however, be kept in mind that inferring the neighborhood effect solely on the basis of the neighbor-dependence probability might bias the results due to the fact that a high proportion of reactions involved in central metabolism of an organism will always be present due to their functional importance. Using ortholog and synteny data in conjunction with neighbor-dependence probability would lead to better inference of the role of network structure on metabolic evolution. The idea being that if a reaction is present in most of the species that are evolutionarily close to the one being considered then it has a higher chance of being added, and if it is genetically linked to other reactions then they have a greater chance of being consecutively added or deleted.

The neighbor-dependent model [9] and the hybrid model described here define reaction neighborhood as reactions sharing at least one metabolite. Alternate definitions of reaction neighborhood are also possible. For example, one possible alternate is to consider the reaction directions when calculating the neighborhood and to regard two reactions as neighbors only if the metabolite connecting the two reactions is a substrate of one and the product of the other. Similarly, it is also possible to use other measures such as sequence similarity [27]–[29] or network distance measures [30], [31] in conjunction with the network structure to model the evolution of metabolic networks. There are, however, limitations associated with the models of metabolic evolution solely based on network structure and sequence similarity. There are a number of other factors affecting metabolic evolution. These include substrate availability (for example, availability of a new nutrient in the environment may favor the insertion of reactions which bring this new metabolite into the mainstream metabolism), gene expression (for example, a decrease in the gene expression relating to an enzyme catalyzing a reaction may force the metabolic network to find alternate routes) and reaction mechanism (for example, a reaction which is chemically inefficient may be favored for deletion compared to an efficient reaction). Factors such as these must be taken into account when modeling the evolution of metabolic networks to depict a more realistic picture of evolution.

We also presented a Gibbs sampler to sample the networks at internal nodes of a phylogenetic tree where the internal networks were sampled by conditioning on three neighbors (one parent and two children) in an approach similar to the one used by Holmes and Bruno [18] for DNA sequence alignment. The sampler considered the network structure surrounding the hyperedge being sampled in addition to the state of the hyperedge in the three neighboring networks when calculating the new state thus resulting in an informed sampling procedure. When sampling ancestral networks, it was assumed that all sampled networks were valid networks. However, not all networks may be functionally viable. For example, a network might not be able to produce a key metabolite which is required or may result in disconnected components that compromise network functionality. Checking for validity of networks occurring at ancestral nodes is an important area for further research.

A Gibbs sampler to estimate the evolution parameters was also presented. Standard distributions were used to generate proposals for parameters. The standard distributions provide satisfactory mixing of the MCMC sampler with appropriate scaling [32]. The rate parameters were sampled from a gamma distribution where scale and shape parameters were calculated from the current network and the proposals for neighbor dependence probability were generated using a beta distribution with its scale parameters calculated from the networks present at the leaves of the given phylogenetic tree. A uniform prior was used when estimating the parameters, which assigns equal probability to each point in the parameter space. It might be useful to explore the dependence between the number of insertions and deletions on the given phylogeny and to investigate the use of other prior distributions. Besides this, when calculating the likelihood of evolution, it was assumed that the phylogenetic tree was known through sequence analysis. This simplified the problem by not requiring a sum over all possible branch lengths. However, when calculating the tree using sequence data, the branch lengths depend on the set of genes used for generating the tree and their evolutionary distances. Thus, different set of genes used could result in different branch lengths. To be able to make useful inferences using an evolutionary model such as the one described here, this uncertainty in the tree must be taken into account by summing over all possible branch lengths. In addition, the effects of using a phylogenetic tree constructed *de novo* from metabolic networks [30], [31] on the model need to be further explored.

The evolution parameters were estimated on a phylogeny connecting the metabolic networks of bacteria belonging to the genus *Pseudomonas* using the Gibbs sampler. The likelihood values for reactions to be present at various levels of the *Pseudomonas* phylogeny were also calculated using the networks visited by the Gibbs sampler and the results were compared to those obtained using parsimony. The stochastic assignment of reactions in ancestral networks offers an edge over deterministic approaches like parsimony which provides the minimum number of transformations required to explain the evolution of a reaction of the tree and can, therefore, underestimate the total number of changes. In addition, using the MCMC approach based on neighbor dependence takes network structure into account, and may be particularly useful in resolving ancestral predictions at the root of phylogenies, or in situations where parsimony is unable to assign states unambiguously (see Figures 7 and 8).

An important factor affecting the results when estimating the evolution parameters and reconstructing the ancestral networks relates to the use of individual pathway maps. Although computationally tractable, individual pathway maps do not take a complete network perspective and may, therefore, lead to incorrect results by ignoring a part of the reaction neighborhood, the so-called border effect. This is particularly true for reactions which occur at the boundary of a metabolic pathway map, which may have a large number of their neighbors not included in that pathway map. The calculation of reaction neighborhood solely using the pathway map under consideration ignores all neighboring reactions that are not present in the pathway map thus affecting the likelihood values. For example, consider R01424 in phenylalanine metabolism. This reaction is present in thirteen out of seventeen pseudomonads including all three *P. syringae* strains, all four *P. putida* strains and two out of the three *P. fluorescens* strains. It was, therefore, expected that the reaction would have a high likelihood of being present in the common ancestor of *P. fluorescens*, *P. syringae* and *P. putida* but, on contrary, was reported to have a relatively low likelihood at this level (Figure 11). Closer inspection of the reaction revealed that it links the phenylalanine metabolism to the pathway map relating to benzoate degradation via coenzyme A and has neighbors spanning across multiple pathway maps. Phenylalanine pathway map contains only 2 neighboring reactions of the reaction R01424 whereas using the data from all pathway maps relating to metabolism results in 53 neighbors. Thus, evaluating the likelihood of R01424 at ancestral levels of the *Pseudomonas* phylogeny solely on the basis of reactions involved in phenylalanine metabolism leads to a very poor neighborhood surrounding the reaction and, consequently, weights down the presence of the reaction in the common ancestor resulting in a low likelihood value. A possible solution to overcome this border effect is to use the full network structure when calculating reaction neighborhoods. However, the computational feasibility of using full network structure when calculating reaction neighborhoods requires further investigation.

When performing the analyses the hyperedges present in all seventeen *Pseudomonas* strains were defined as core and the hyperedges missing from all the strains were defined as prohibited hyperedges. However, the results presented in this analysis suggest that pathogenic bacteria belonging to species *P. syringae* have gone through a high number of deletion events compared to other species. Assigning core edges solely on the basis of intersection model may, therefore, bias the results towards the loss of reactions which might be essential in non-pathogenic bacteria. Similarly, prohibiting reactions that are not present in any one of the seventeen genome-sequenced strains would prevent the common ancestor from having reactions which might have been lost very early during the course of evolution. To model scenarios like these the provision of having a lineage specific core and prohibited hyperedges must be explored. Alternatively, it might be useful to assign core and prohibited hyperedges using the ortholog data from closely related bacteria, or by incorporating metabolic information from organisms sharing the same environment in the set of permitted reactions. Comparing ancestral network predictions generated using different set of core and prohibited hyperedges might provide clues about the functionality of the common ancestors of the bacteria and the environment the ancestors might have colonized.

Finally, the analysis presented here uses data from the KEGG database. The metabolic annotations available for the majority of genome-sequenced organisms are generated using automated annotation tools based on the similarity of predicted genes to genes of known function and therefore contain a substantial amount of noise. For example, some genes predicted to have a broad enzymatic function are linked to multiple reactions, while others fail to meet the detection threshold for annotation and are therefore recorded as absent. Nevertheless, networks deposited in databases like KEGG are commonly treated as if they are as certain as sequence data, which is a serious error that undermines many present investigations. It would be desirable to take this noise into account while modeling the evolution of metabolic networks. One way would be to use hidden states to model experimentally validate metabolisms which are observed though predicted metabolisms. This will not only enable one to model the noise in the data but also allow correct prediction of a metabolism for an organism using homologous information similar to comparative genome annotation [33].

In summary, evolutionary modeling of metabolic network is an important area. Using statistical models of network evolution such as the one described here not only allow one to investigate how the metabolic networks evolve in closely related organisms but also enable testing of biological hypotheses such as specialization of genomes and identification of regions of metabolic networks that are under high selection.

## Supporting Information

Figure S1 Simulation results for insertion frequencies for the toy network H_1_ shown in Figure 1 using hybrid model for different values of δ. Also shown in the top panel are the number of neighbors for each hyperedge based on the reference network.
(0.02 MB PDF)

Figure S2 Sub-networks of the toy network H_1_ shown in Figure 1 for different sub-network sizes (N) obtained by iteratively dividing the network on the basis of neighborhood. The hyperedges which were originally absent from H_1_ but present in the sub-network are shown in gray.
(0.05 MB PDF)

Figure S3 Likelihood surfaces calculated by matrix exponentiation using all 1024 networks (True Likelihood) and using the networks visited by the Gibbs sampler (Estimated Likelihood) for different insertion and deletion rates for the toy networks phylogeny shown in Figure 1. The true and estimated maximum likelihood values are marked with asterisks. The maximum likelihood value was estimated using the Gibbs sampler for parameter estimation.
(0.11 MB PDF)

Figure S4 An example MCMC trace showing the rate parameters for the first 1,000 iterations of the Gibbs sampler for the toy networks phylogeny shown in Figure 1.
(0.04 MB PDF)

Figure S5 Example MCMC traces showing the rate parameters for the first 1,000 iterations of the Gibbs sampler initiated from different starting values. The sampler was run on the *Pseudomonas fluorescens* phylogeny shown in Figure 6B for different metabolic networks.
(0.22 MB PDF)

Figure S6 Number of insertion and deletion events for the alterable reactions, that is the reactions which were neither defined as core nor were defined as prohibited in the network obtained using the Gibbs sampler run under the hybrid model. The sampler was run for the six pathway maps used in this analysis over the phylogeny connecting the seventeen *Pseudomonas* strains shown in Figure 6A for 110,000 iterations with the first 10,000 iterations regarded as burn-in period. Samples were collected every 10^th^ iteration.
(0.02 MB PDF)

Figure S7 Number of insertion and deletion events at each branch of the phylogeny connecting the seventeen *Pseudomonas* strains shown in Figure 6A obtained using the Gibbs sampler run under the hybrid model. The sampler was run for 110,000 iterations with the first 10,000 iterations regarded as burn-in period. Samples were collected every 10^th^ iteration. Strain abbreviations: pae: *P. aeruginosa* PAO1, pap: *P. aeruginosa* PA7, pau: *P. aeruginosa* PA14, pag: *P. aeruginosa* LESB58, pen: *P. entomophila* L48, pfl: *P. fluorescens* Pf-5, pfo: *P. fluorescens* Pf0-1, pfs: *P. fluorescens* SBW25, pmy: *P. mendocina* ymp, ppf: *P. putida* F1, ppg: *P. putida* GB-1 ppu: *P. putida* KT2440, ppw: *P. putida* W619, psa: *P. stutzeri* A1501, psb: *P. syringae* pv. syringae B728a, psp: *P. syringae* pv. phaseolicola 1448A, and pst: *P. syringae* pv. tomato DC3000.
(0.02 MB PDF)

Figure S8 Degree distributions of nodes at the ancestral levels of the *Pseudomonas* phylogney shown in Figure 6A for the glycolysis/gluconeogenesis map obtained using the Gibbs sampler. The actual degree distributions observed for the seventeen genome-sequenced *Pseudomonas* strains are shown in red. Strain abbreviations: pae: *P. aeruginosa* PAO1, pap: *P. aeruginosa* PA7, pau: *P. aeruginosa* PA14, pag: *P. aeruginosa* LESB58, pen: *P. entomophila* L48, pfl: *P. fluorescens* Pf-5, pfo: *P. fluorescens* Pf0-1, pfs: *P. fluorescens* SBW25, pmy: *P. mendocina* ymp, ppf: *P. putida* F1, ppg: *P. putida* GB-1 ppu: *P. putida* KT2440, ppw: *P. putida* W619, psa: *P. stutzeri* A1501, psb: *P. syringae* pv. syringae B728a, psp: *P. syringae* pv. phaseolicola 1448A, and pst: *P. syringae* pv. tomato DC3000.
(0.04 MB PDF)

Figure S9 Degree distributions of nodes at the ancestral levels of the *Pseudomonas* phylogney shown in Figure 6A for the pentose phosphate pathway map obtained using the Gibbs sampler. The actual degree distributions observed for the seventeen genome-sequenced *Pseudomonas* strains are shown in red. Strain abbreviations: pae: *P. aeruginosa* PAO1, pap: *P. aeruginosa* PA7, pau: *P. aeruginosa* PA14, pag: *P. aeruginosa* LESB58, pen: *P. entomophila* L48, pfl: *P. fluorescens* Pf-5, pfo: *P. fluorescens* Pf0-1, pfs: *P. fluorescens* SBW25, pmy: *P. mendocina* ymp, ppf: *P. putida* F1, ppg: *P. putida* GB-1 ppu: *P. putida* KT2440, ppw: *P. putida* W619, psa: *P. stutzeri* A1501, psb: *P. syringae* pv. syringae B728a, psp: *P. syringae* pv. phaseolicola 1448A, and pst: *P. syringae* pv. tomato DC3000.
(0.07 MB PDF)

Figure S10 Degree distributions of nodes at the ancestral levels of the *Pseudomonas* phylogney shown in Figure 6A for the lysine degradation map obtained using the Gibbs sampler. The actual degree distributions observed for the seventeen genome-sequenced *Pseudomonas* strains are shown in red. Strain abbreviations: pae: *P. aeruginosa* PAO1, pap: *P. aeruginosa* PA7, pau: *P. aeruginosa* PA14, pag: *P. aeruginosa* LESB58, pen: *P. entomophila* L48, pfl: *P. fluorescens* Pf-5, pfo: *P. fluorescens* Pf0-1, pfs: *P. fluorescens* SBW25, pmy: *P. mendocina* ymp, ppf: *P. putida* F1, ppg: *P. putida* GB-1 ppu: *P. putida* KT2440, ppw: *P. putida* W619, psa: *P. stutzeri* A1501, psb: *P. syringae* pv. syringae B728a, psp: *P. syringae* pv. phaseolicola 1448A, and pst: *P. syringae* pv. tomato DC3000.
(0.08 MB PDF)

Figure S11 Degree distributions of nodes at the ancestral levels of the *Pseudomonas* phylogney shown in Figure 6A for the histidine metabolism map obtained using the Gibbs sampler. The actual degree distributions observed for the seventeen genome-sequenced *Pseudomonas* strains are shown in red. Strain abbreviations: pae: *P. aeruginosa* PAO1, pap: *P. aeruginosa* PA7, pau: *P. aeruginosa* PA14, pag: *P. aeruginosa* LESB58, pen: *P. entomophila* L48, pfl: *P. fluorescens* Pf-5, pfo: *P. fluorescens* Pf0-1, pfs: *P. fluorescens* SBW25, pmy: *P. mendocina* ymp, ppf: *P. putida* F1, ppg: *P. putida* GB-1 ppu: *P. putida* KT2440, ppw: *P. putida* W619, psa: *P. stutzeri* A1501, psb: *P. syringae* pv. syringae B728a, psp: *P. syringae* pv. phaseolicola 1448A, and pst: *P. syringae* pv. tomato DC3000.
(0.04 MB PDF)

Figure S12 Degree distributions of nodes at the ancestral levels of the *Pseudomonas* phylogney shown in Figure 6A for the phenylalanine metabolism map obtained using the Gibbssampler. The actual degree distributions observed for the seventeen genome-sequenced *Pseudomonas* strains are shown in red. Strain abbreviations: pae: *P. aeruginosa* PAO1, pap: *P. aeruginosa* PA7, pau: *P. aeruginosa* PA14, pag: *P. aeruginosa* LESB58, pen: *P. entomophila* L48, pfl: *P. fluorescens* Pf-5, pfo: *P. fluorescens* Pf0-1, pfs: *P. fluorescens* SBW25, pmy: *P. mendocina* ymp, ppf: *P. putida* F1, ppg: *P. putida* GB-1 ppu: *P. putida* KT2440, ppw: *P. putida* W619, psa: *P. stutzeri* A1501, psb: *P. syringae* pv. syringae B728a, psp: *P. syringae* pv. phaseolicola 1448A, and pst: *P. syringae* pv. tomato DC3000.
(0.03 MB PDF)

Figure S13 Degree distributions of nodes at the ancestral levels of the *Pseudomonas* phylogney shown in Figure 6A for the pyruvate metabolism map obtained using the Gibbs sampler. The actual degree distributions observed for the seventeen genome-sequenced *Pseudomonas* strains are shown in red. Strain abbreviations: pae: *P. aeruginosa* PAO1, pap: *P. aeruginosa* PA7, pau: *P. aeruginosa* PA14, pag: *P. aeruginosa* LESB58, pen: *P. entomophila* L48, pfl: *P. fluorescens* Pf-5, pfo: *P. fluorescens* Pf0-1, pfs: *P. fluorescens* SBW25, pmy: *P. mendocina* ymp, ppf: *P. putida* F1, ppg: *P. putida* GB-1 ppu: *P. putida* KT2440, ppw: *P. putida* W619, psa: *P. stutzeri* A1501, psb: *P. syringae* pv. syringae B728a, psp: *P. syringae* pv. phaseolicola 1448A, and pst: *P. syringae* pv. tomato DC3000.
(0.08 MB PDF)

Table S1 Pseudo-likelihood P^*^(H_2_|H_1_) conditioned on H for the toy networks shown in Figure 1 using different sub-network sizes. Also shown are the exact values calculated by full network exponentiation and by using the MCMC approach based on path sampling described in Mithani *et al.* [9] with 11,000 iterations including the first 1,000 iteration as burn-in period.
(0.03 MB PDF)

Table S2 Basic information of the metabolic networks for the seventeen genome-sequenced strains of *Pseudomonas* used in this study. A reversible reaction was represented by two hyperedges (one in either direction) in this study. The codes MAPxxxxx correspond to the respective KEGG pathway codes [22].
(0.04 MB PDF)

Table S3 Effective sample sizes (ESS) for the estimated parameters (δ: neighbor dependence probability, λ: insertion rate and μ: deletion rate) using the Gibbs sampler run under the hybrid model for the evolution of metabolic networks over the phylogeny connecting different *Pseudomonas* strains (Figure 6). Hyperedges that were common to all seventeen strains were regarded as core edges and hyperedges missing in all seventeen strains were regarded as prohibited edges. The values are averaged across three runs of 60,000 iterations for *P. fluorescens* and *P. syringae* phylogenies, and 110,000 iterations for the phylogeny connecting the seventeen *Pseudomonas* strains with the first 10,000 iterations regarded as burn-in period in each case. Samples were collected every 10^th^ iteration. Strain abbreviations: pfl: *Pseudomonas fluorescens* Pf-5, pfo: *Pseudomonas fluorescens* Pf0-1, pfs: *Pseudomonas fluorescens* SBW25, psb: *Pseudomonas syringae* pv. syringae B728a, psp: *Pseudomonas syringae* pv. phaseolicola 1448A, and pst: *Pseudomonas syringae* pv. tomato DC3000.
(0.03 MB PDF)

Table S4 Running time and the acceptance percentage of the Gibbs sampler for the estimation of evolution parameters (δ: neighbor dependence probability, λ: insertion rate and μ: deletion rate) run under the hybrid model for different metabolic networks over the phylogeny connecting different *Pseudomonas* strains (Figure 6). Hyperedges that were common to all seventeen strains were regarded as core edges and hyperedges missing in all seventeen strains were regarded as prohibited edges. The values are averaged across three runs of 60,000 iterations for *P. fluorescens* and *P. syringae* phylogenies, and 110,000 iterations for the phylogeny connecting the seventeen *Pseudomonas* strains with the first 10,000 iterations regarded as burn-in period in each case. Samples were collected every 10^th^ iteration. Strain abbreviations: pfl: *Pseudomonas fluorescens* Pf-5, pfo: *Pseudomonas fluorescens* Pf0-1, pfs: *Pseudomonas fluorescens* SBW25, psb: *Pseudomonas syringae* pv. syringae B728a, psp: *Pseudomonas syringae* pv. phaseolicola 1448A, and pst: *Pseudomonas syringae* pv. tomato DC3000.
(0.03 MB PDF)

Text S1 Testing of the Gibbs sampler for parameter estimation.
(0.04 MB PDF)
