# Supplementary figures and images for: A Bayesian Approach to the Evolution of Metabolic Networks on a Phylogeny
Source: PLoS Comput Biol. 2010 Aug 5;6(8):e1000868. doi: 10.1371/journal.pcbi.1000868 (PMC2917375; doi:10.1371/journal.pcbi.1000868)

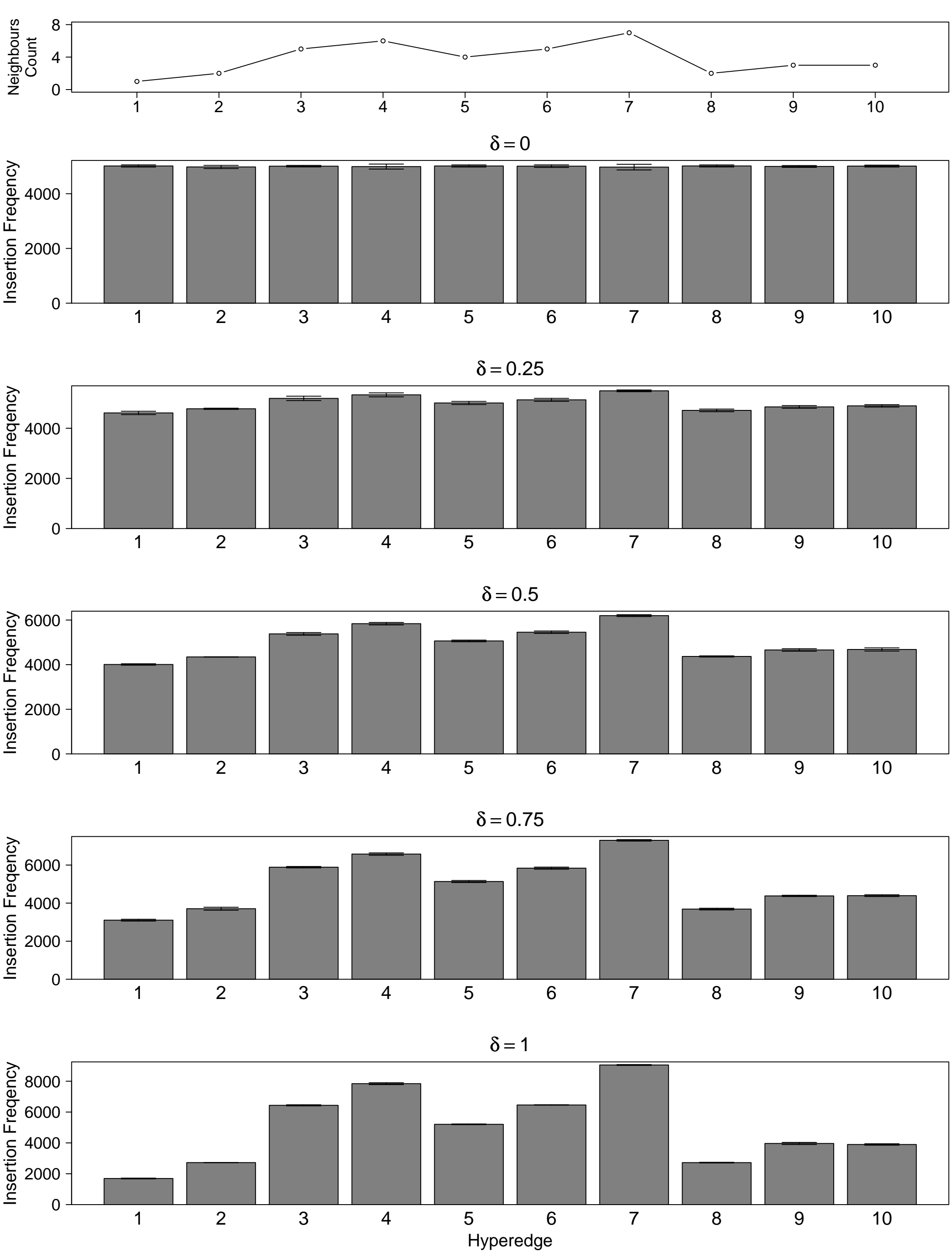

Supplement: Figure S1 — Simulation results for insertion frequencies for the toy network H1 shown in Figure 1 using hybrid model for different values of δ. Also shown in the top panel are the number of neighbors for each hyperedge based on the reference network. (0.02 MB PDF) [file pcbi.1000868.s001.pdf]

**A**    **N = 4**

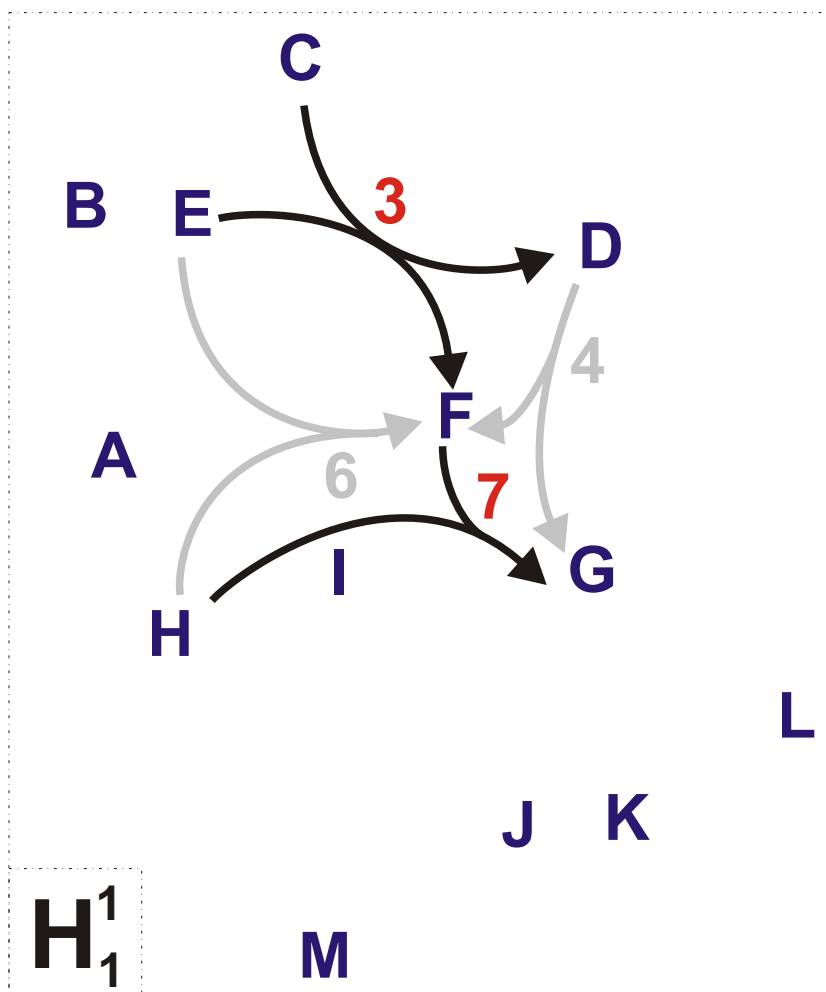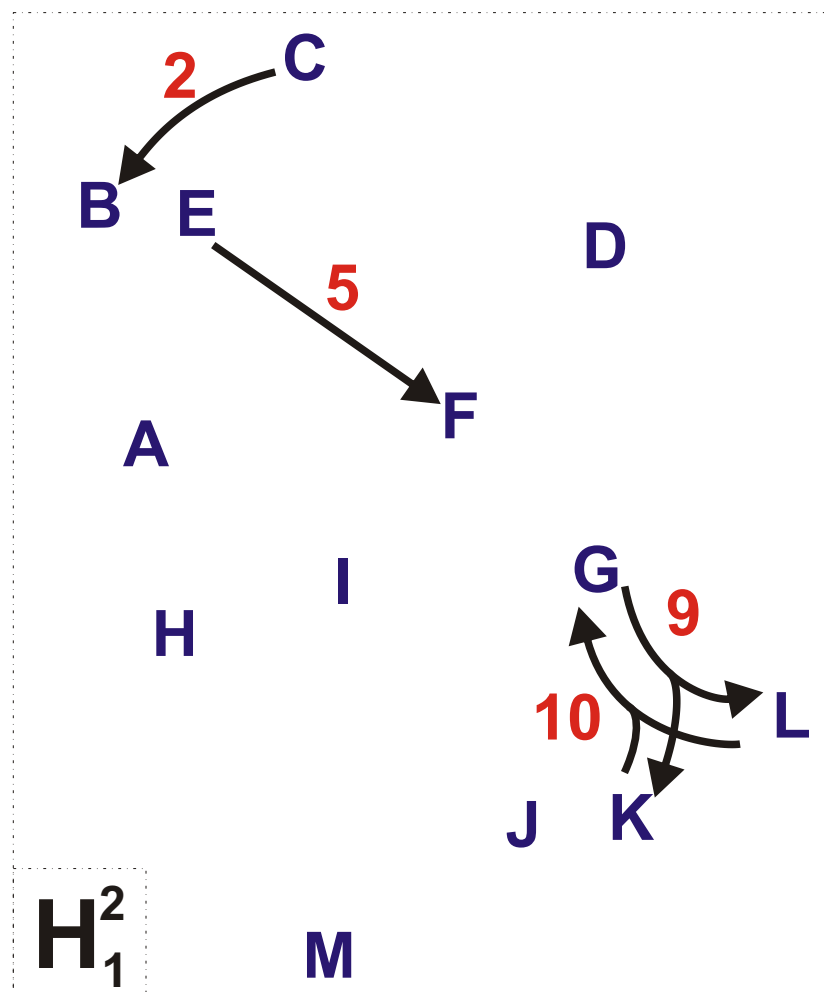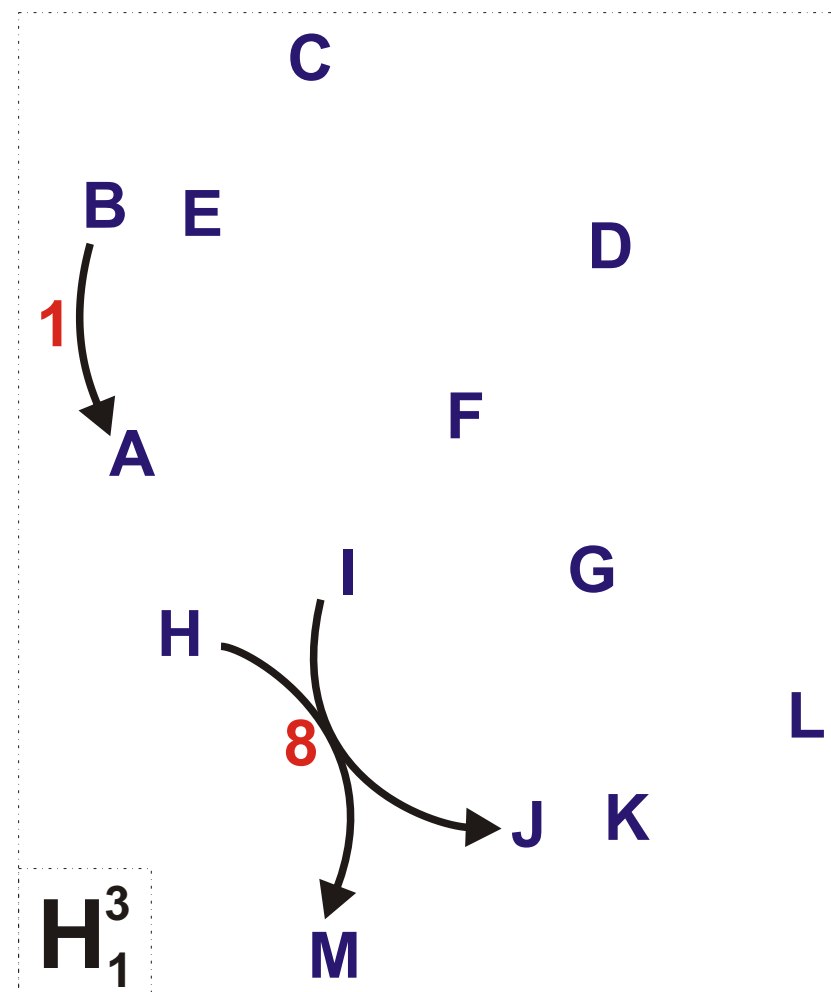

**B**    **N = 5**

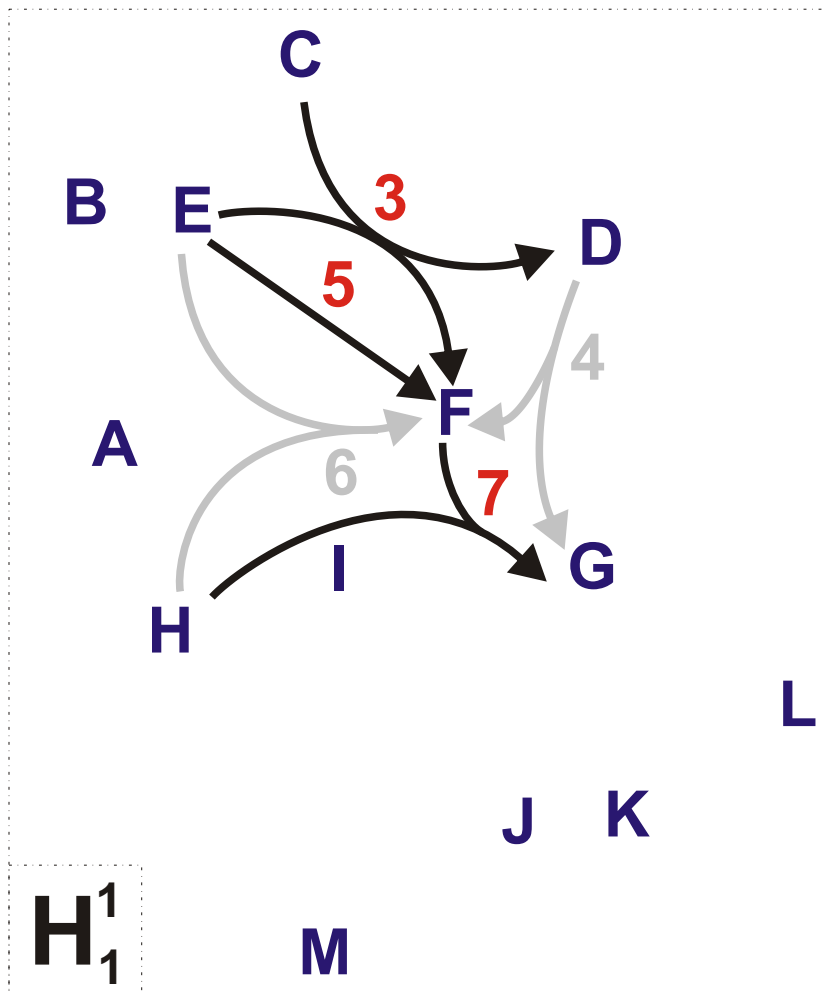

**C**    **N = 6**

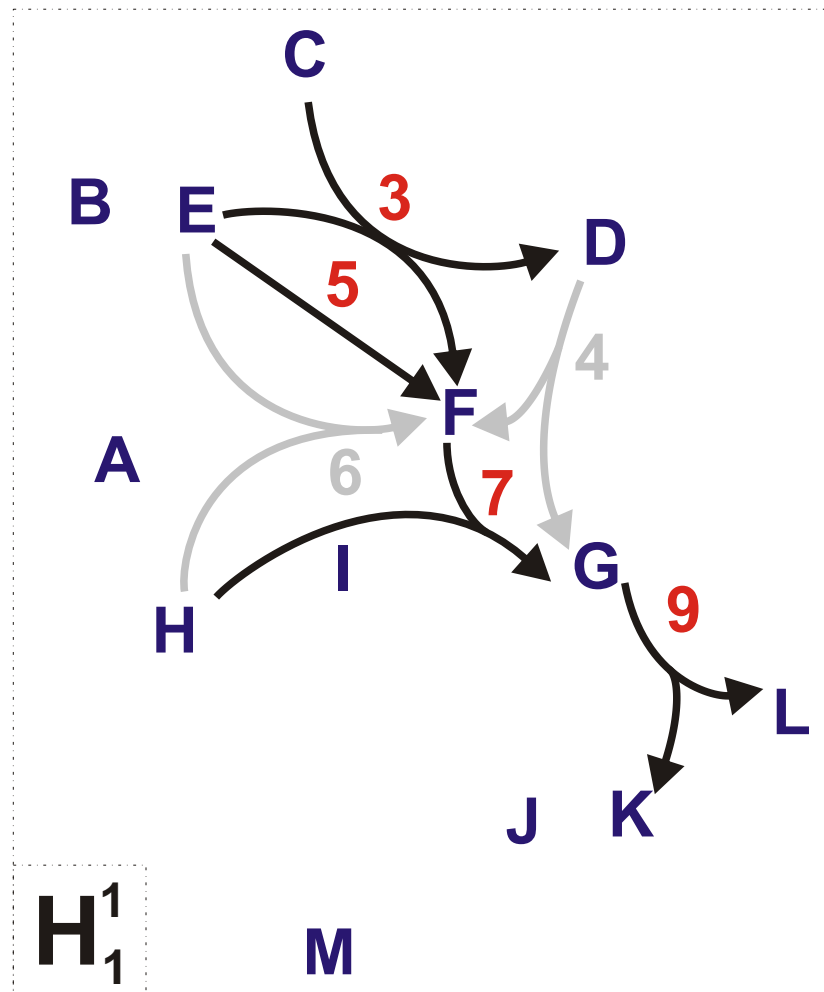

**D**    **N = 7**

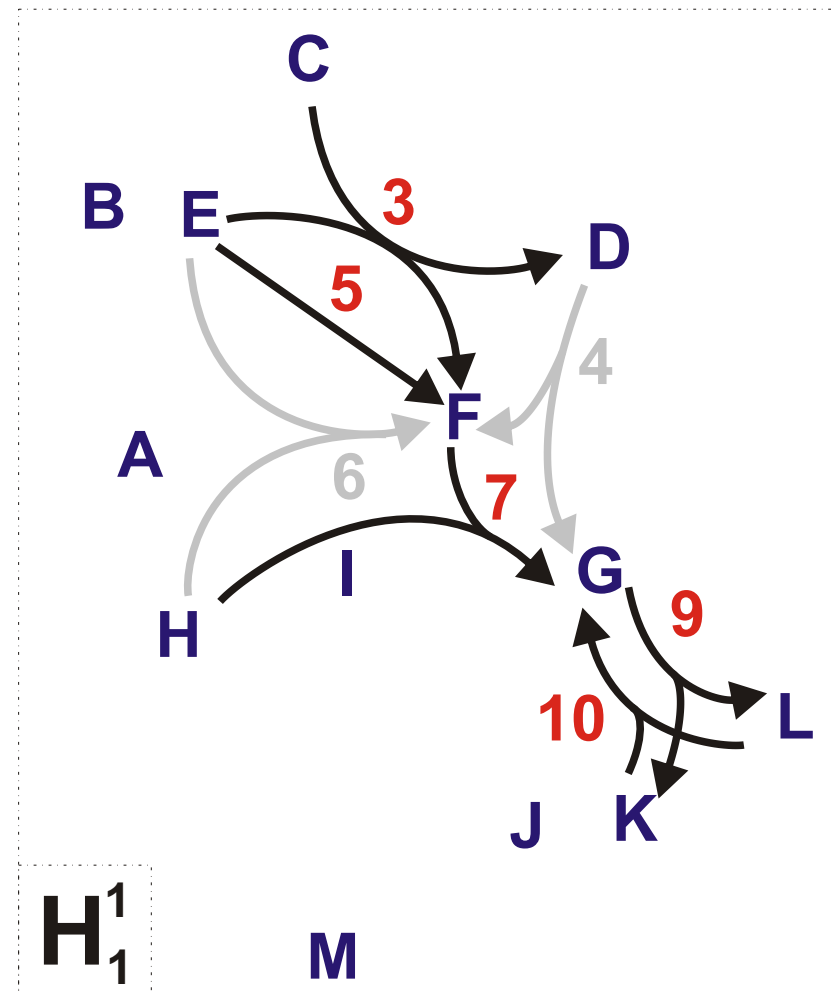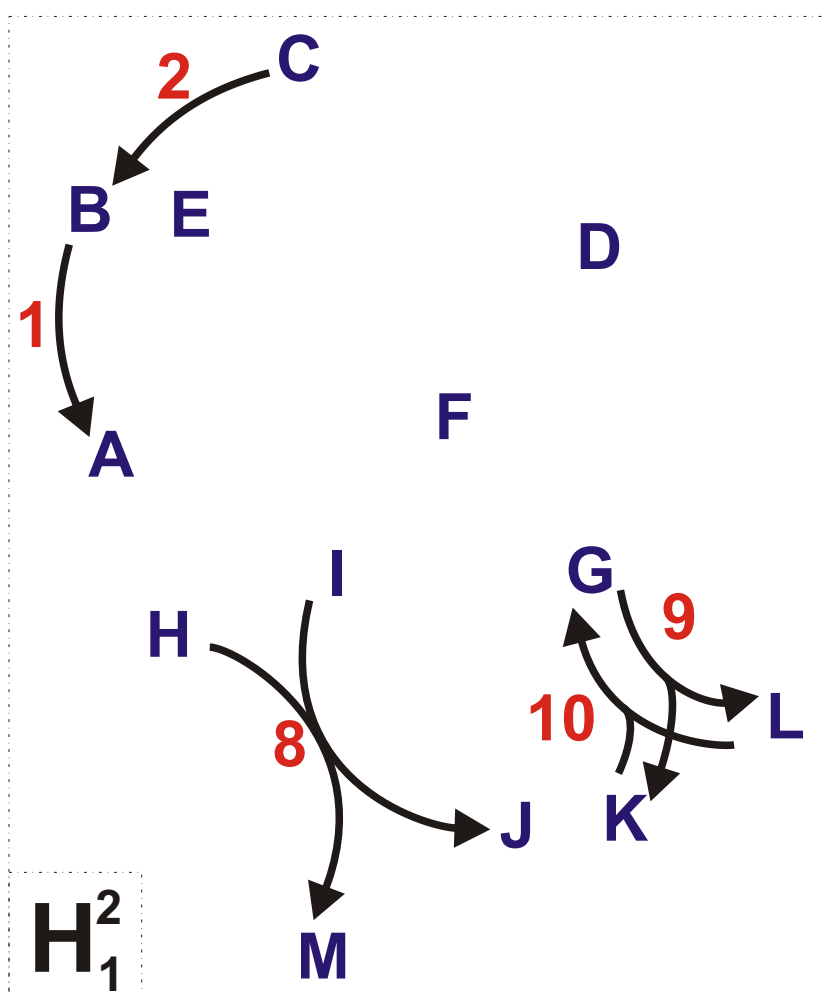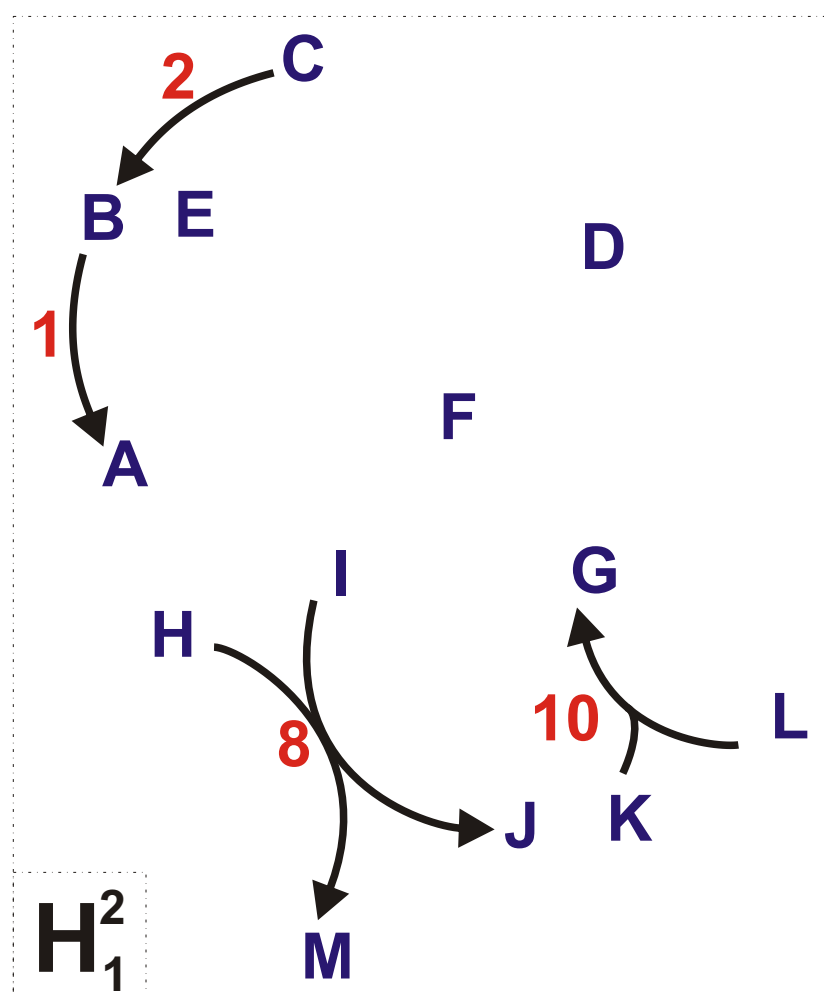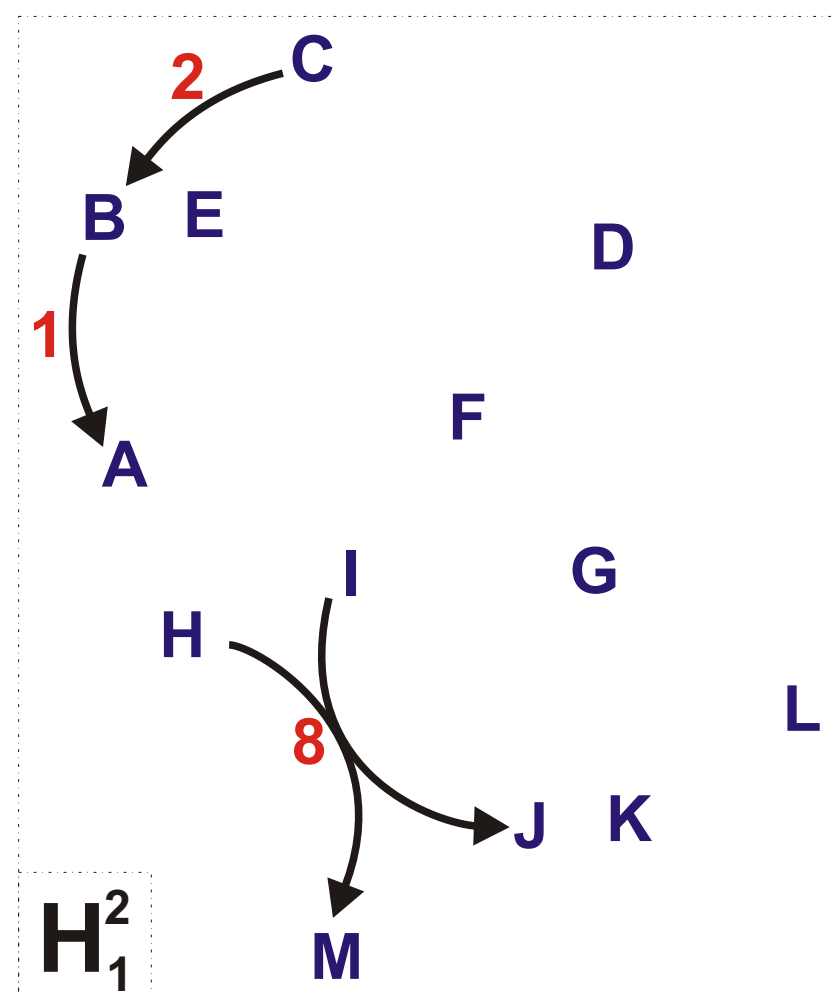

Supplement: Figure S2 — Sub-networks of the toy network H1 shown in Figure 1 for different sub-network sizes (N) obtained by iteratively dividing the network on the basis of neighborhood. The hyperedges which were originally absent from H1 but present in the sub-network are shown in gray. (0.05 MB PDF) [file pcbi.1000868.s002.pdf]

# True Likelihood

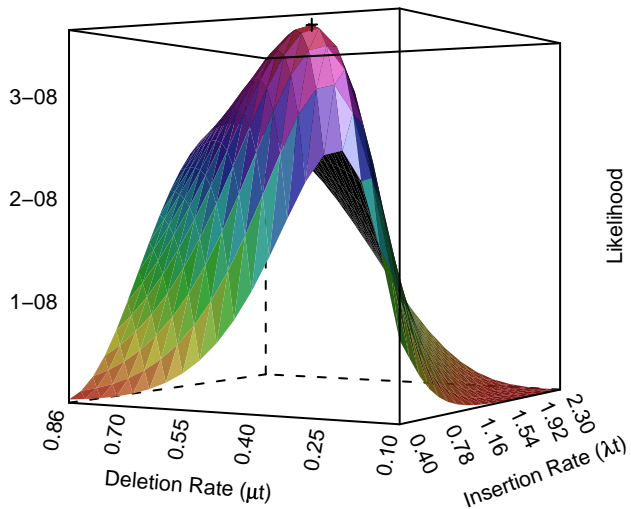

# Estimated Likelihood

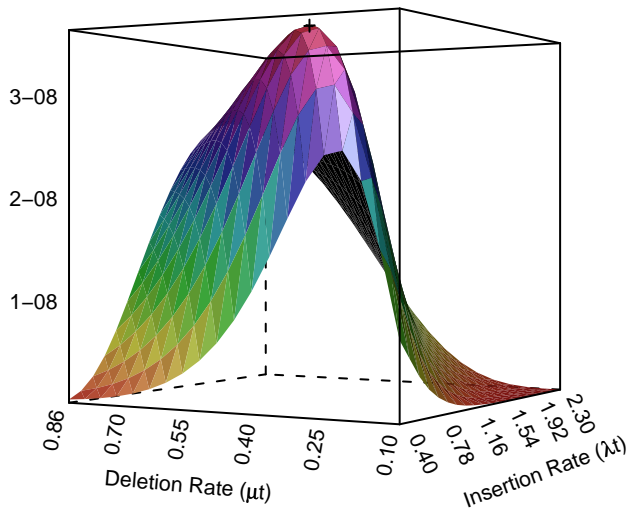

Supplement: Figure S3 — Likelihood surfaces calculated by matrix exponentiation using all 1024 networks (True Likelihood) and using the networks visited by the Gibbs sampler (Estimated Likelihood) for different insertion and deletion rates for the toy networks phylogeny shown in Figure 1. The true and estimated maximum likelihood values are marked with asterisks. The maximum likelihood value was estimated using the Gibbs sampler for parameter estimation. (0.11 MB PDF) [file pcbi.1000868.s003.pdf]

Parameter

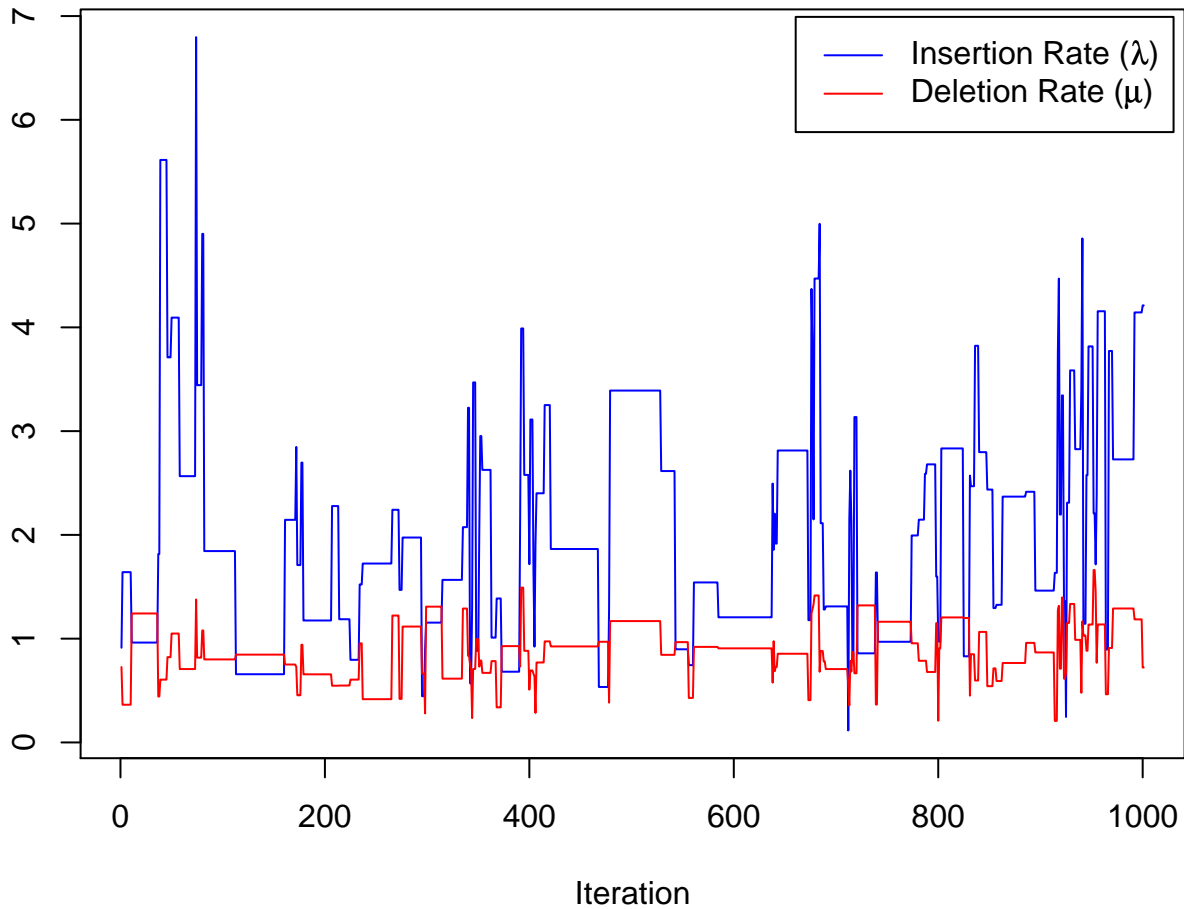

Supplement: Figure S4 — An example MCMC trace showing the rate parameters for the first 1,000 iterations of the Gibbs sampler for the toy networks phylogeny shown in Figure 1. (0.04 MB PDF) [file pcbi.1000868.s004.pdf]

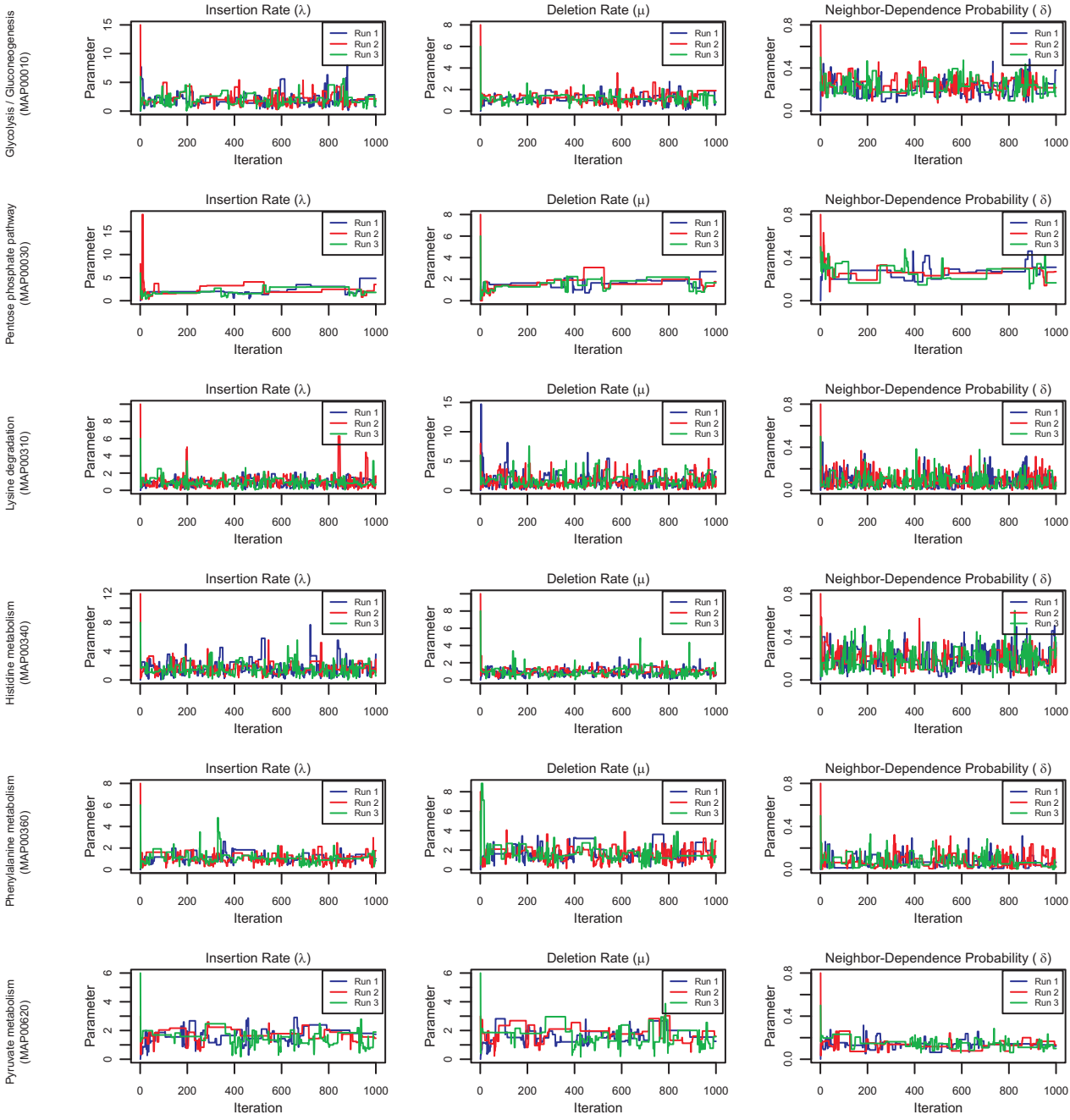

Supplement: Figure S5 — Example MCMC traces showing the rate parameters for the first 1,000 iterations of the Gibbs sampler initiated from different starting values. The sampler was run on the Pseudomonas fluorescens phylogeny shown in Figure 6B for different metabolic networks. (0.22 MB PDF) [file pcbi.1000868.s005.pdf]

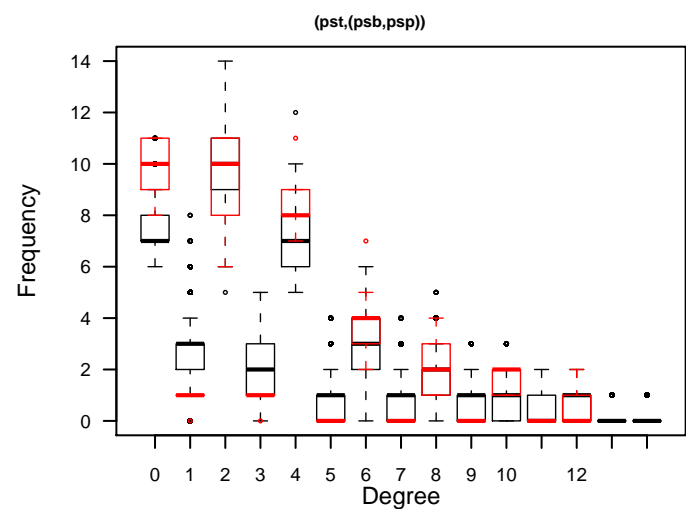

Supplement: Figure S9 — Degree distributions of nodes at the ancestral levels of the Pseudomonas phylogney shown in Figure 6A for the pentose phosphate pathway map obtained using the Gibbs sampler. The actual degree distributions observed for the seventeen genome-sequenced Pseudomonas strains are shown in red. Strain abbreviations: pae: P. aeruginosa PAO1, pap: P. aeruginosa PA7, pau: P. aeruginosa PA14, pag: P. aeruginosa LESB58, pen: P. entomophila L48, pfl: P. fluorescens Pf-5, pfo: P. fluorescens Pf0-1, pfs: P. fluorescens SBW25, pmy: P. mendocina ymp, ppf: P. putida F1, ppg: P. putida GB-1 ppu: P. putida KT2440, ppw: P. putida W619, psa: P. stutzeri A1501, psb: P. syringae pv. syringae B728a, psp: P. syringae pv. phaseolicola 1448A, and pst: P. syringae pv. tomato DC3000. (0.07 MB PDF) [file pcbi.1000868.s009.pdf]

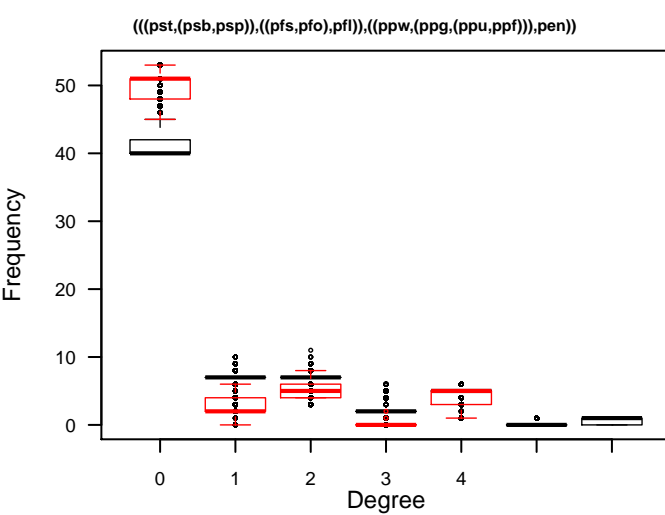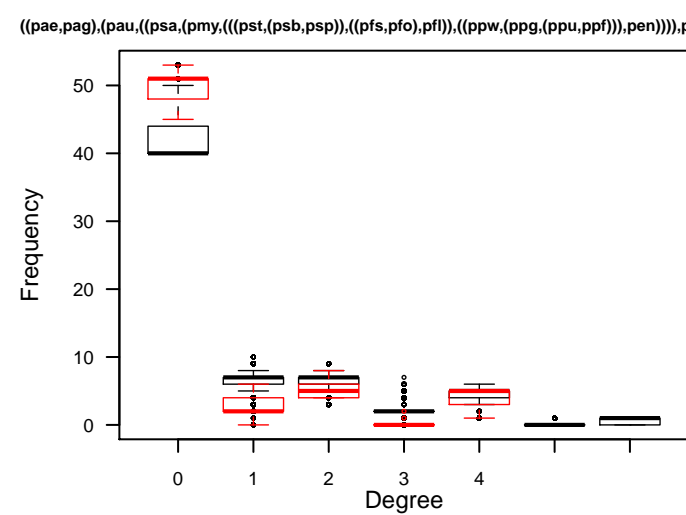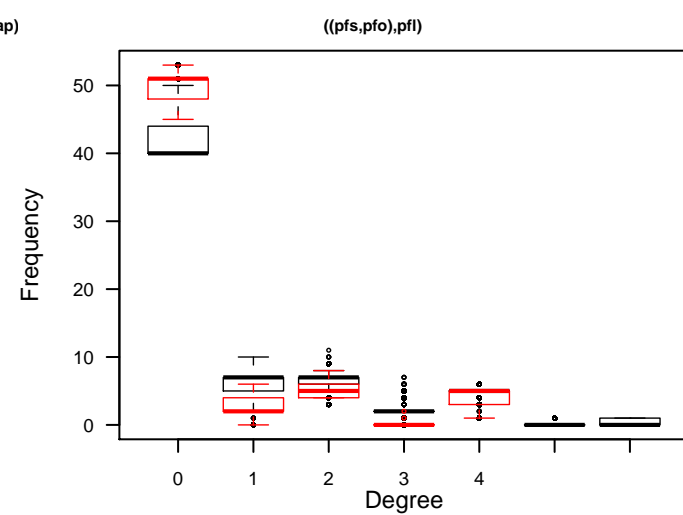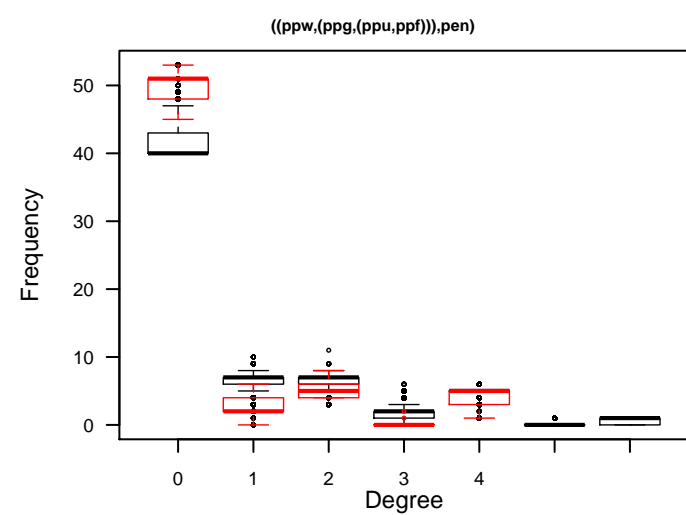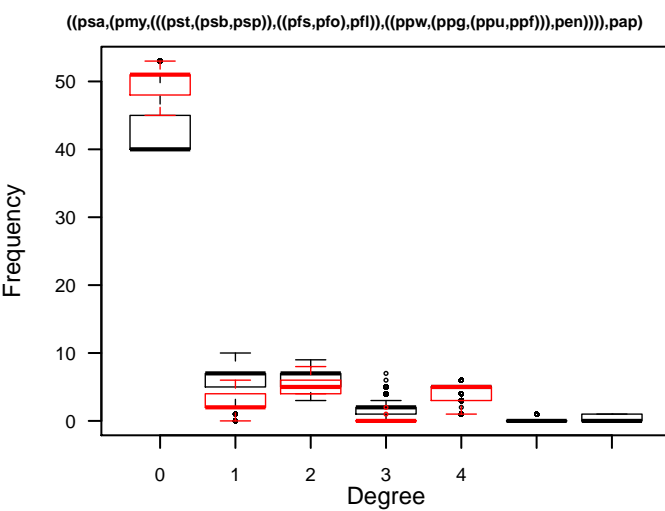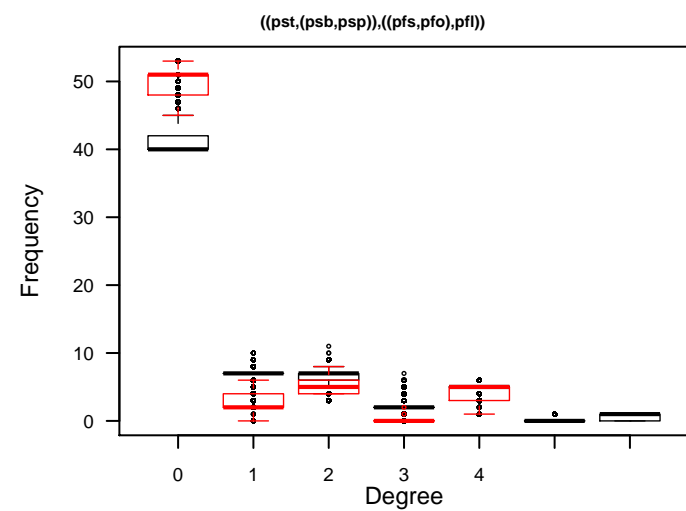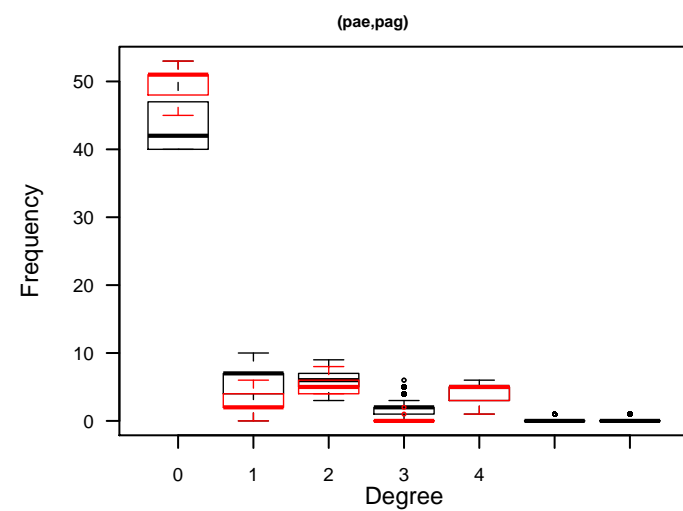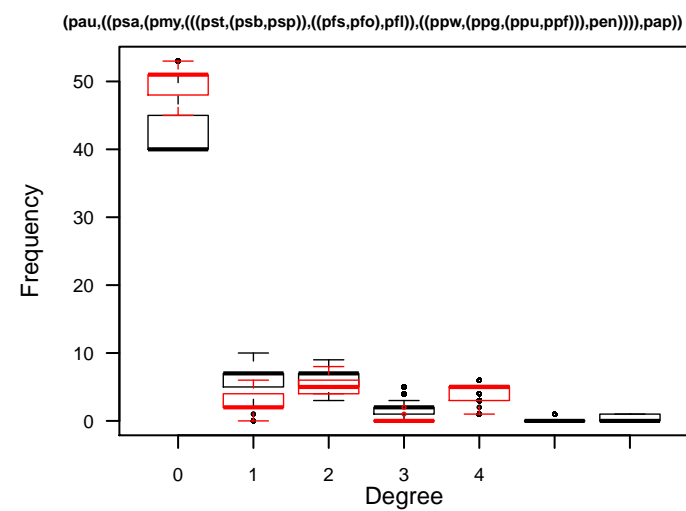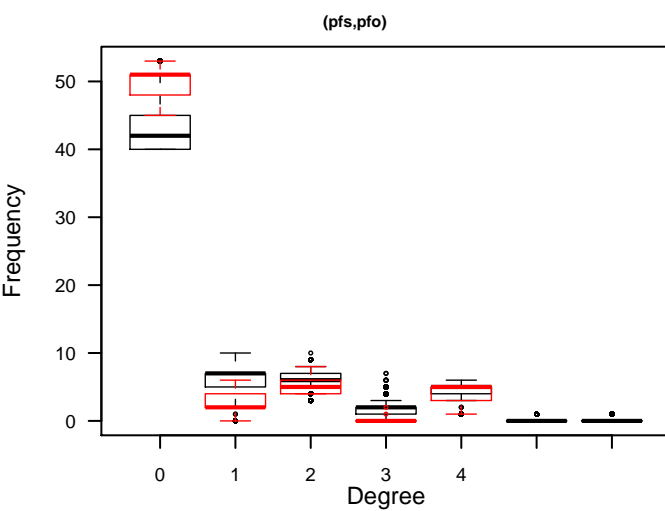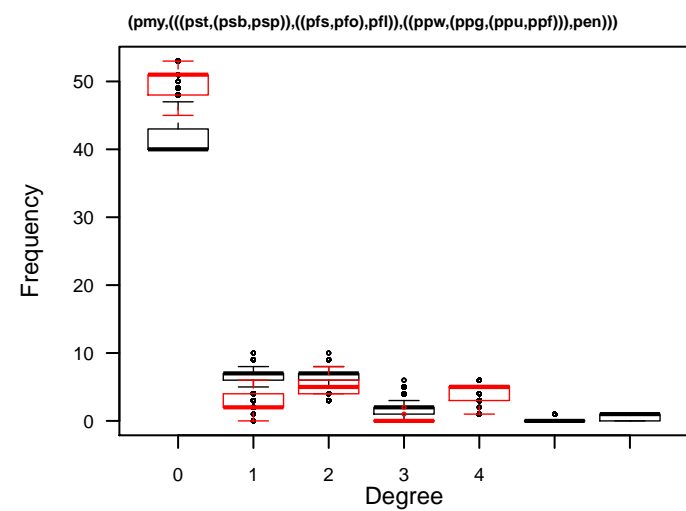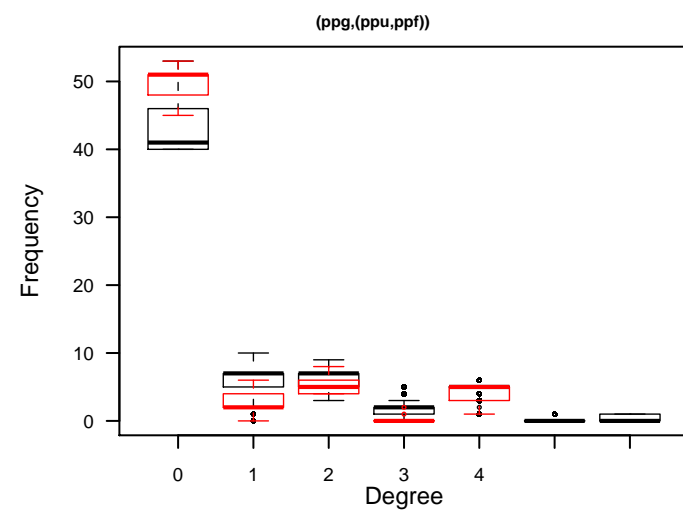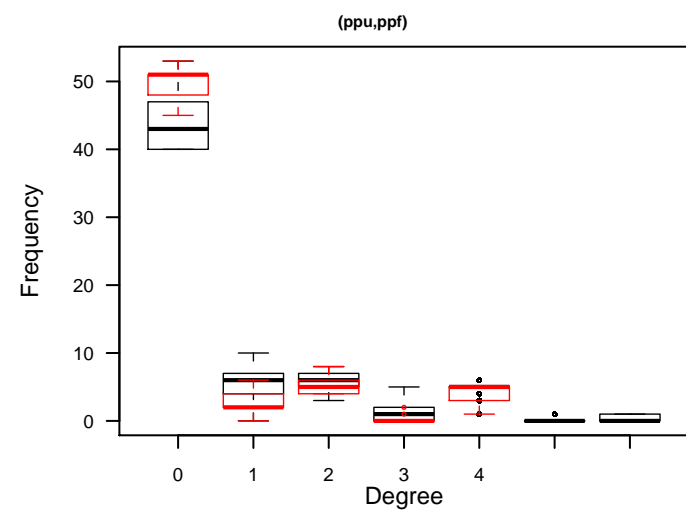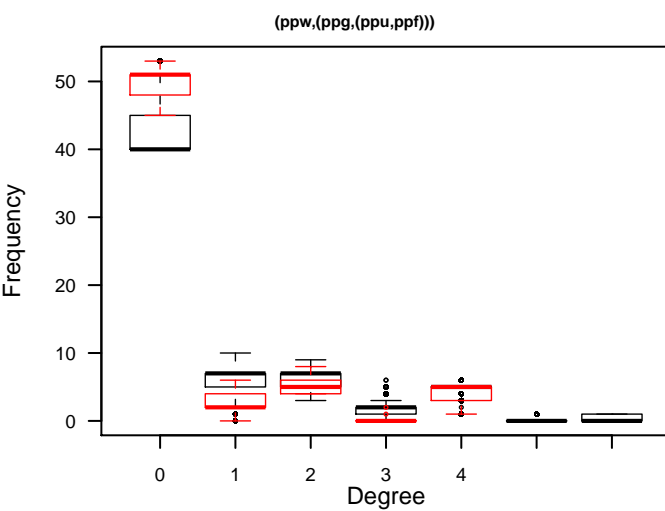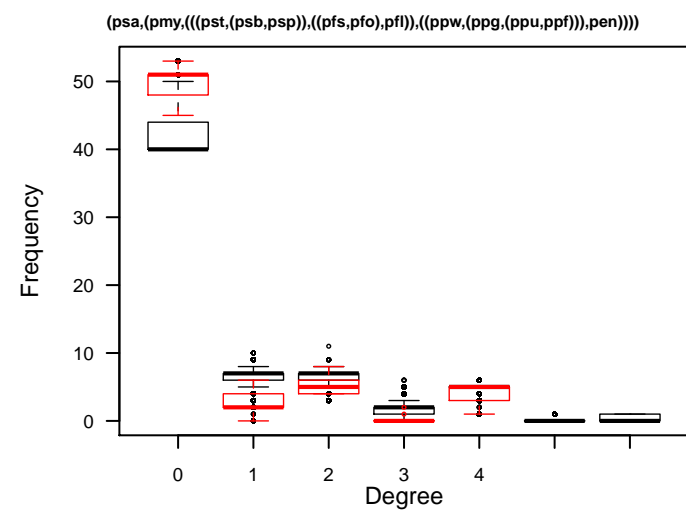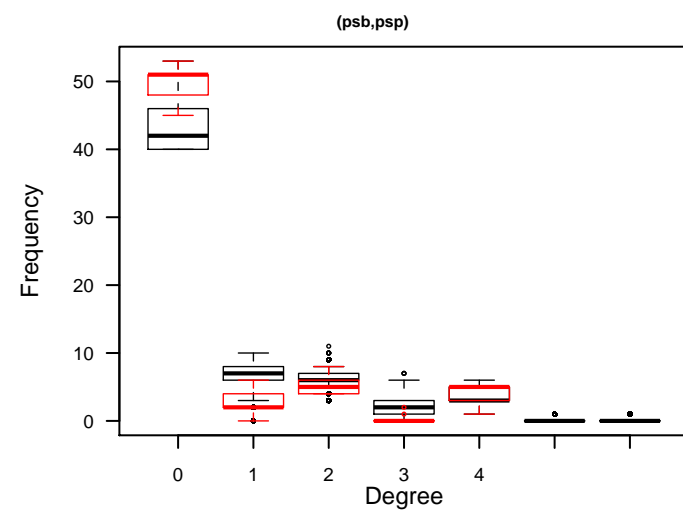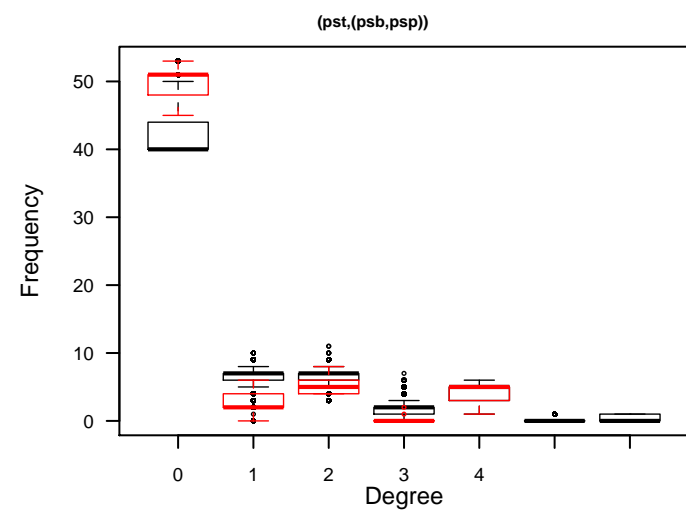

Supplement: Figure S10 — Degree distributions of nodes at the ancestral levels of the Pseudomonas phylogney shown in Figure 6A for the lysine degradation map obtained using the Gibbs sampler. The actual degree distributions observed for the seventeen genome-sequenced Pseudomonas strains are shown in red. Strain abbreviations: pae: P. aeruginosa PAO1, pap: P. aeruginosa PA7, pau: P. aeruginosa PA14, pag: P. aeruginosa LESB58, pen: P. entomophila L48, pfl: P. fluorescens Pf-5, pfo: P. fluorescens Pf0-1, pfs: P. fluorescens SBW25, pmy: P. mendocina ymp, ppf: P. putida F1, ppg: P. putida GB-1 ppu: P. putida KT2440, ppw: P. putida W619, psa: P. stutzeri A1501, psb: P. syringae pv. syringae B728a, psp: P. syringae pv. phaseolicola 1448A, and pst: P. syringae pv. tomato DC3000. (0.08 MB PDF) [file pcbi.1000868.s010.pdf]

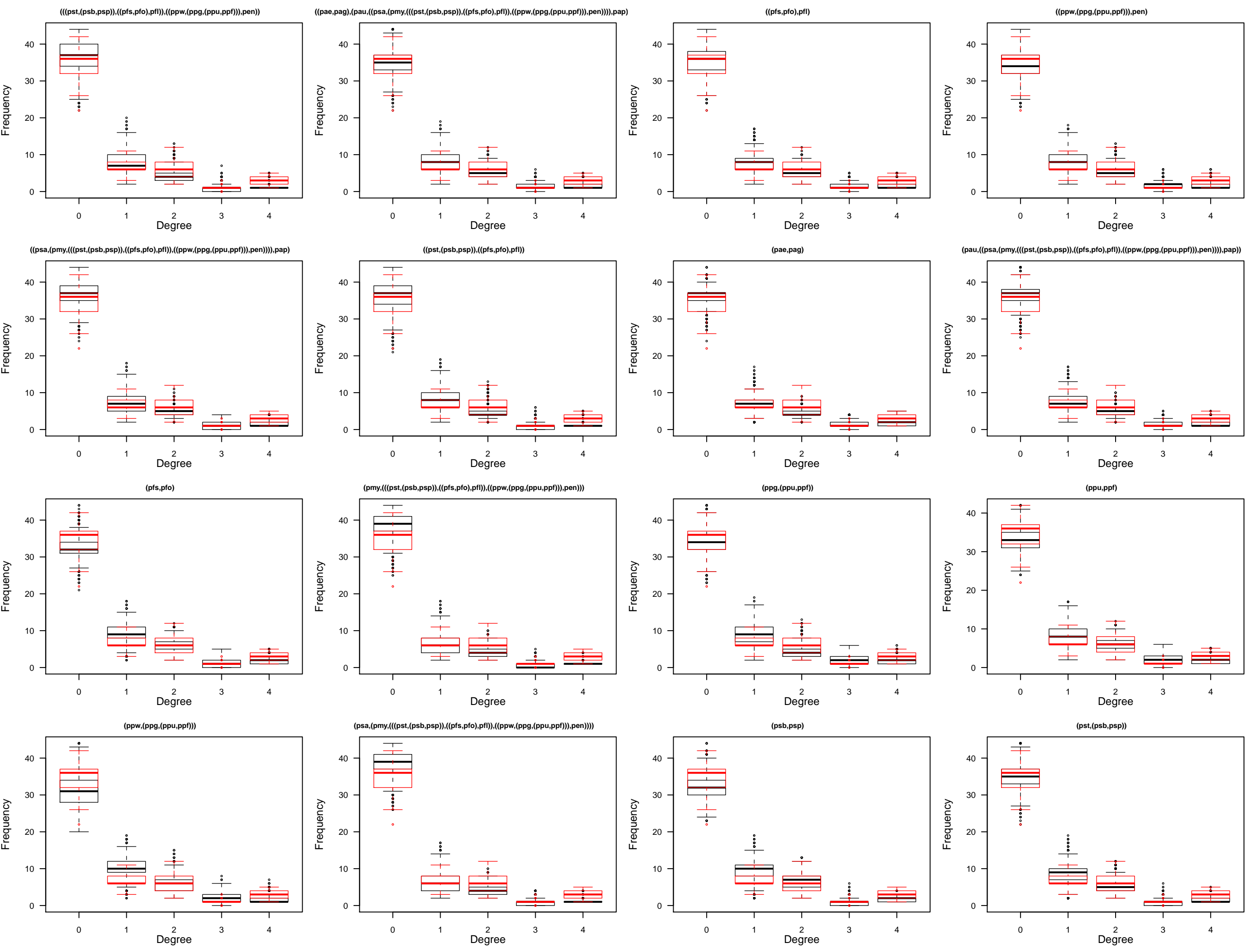

Supplement: Figure S12 — Degree distributions of nodes at the ancestral levels of the Pseudomonas phylogney shown in Figure 6A for the phenylalanine metabolism map obtained using the Gibbssampler. The actual degree distributions observed for the seventeen genome-sequenced Pseudomonas strains are shown in red. Strain abbreviations: pae: P. aeruginosa PAO1, pap: P. aeruginosa PA7, pau: P. aeruginosa PA14, pag: P. aeruginosa LESB58, pen: P. entomophila L48, pfl: P. fluorescens Pf-5, pfo: P. fluorescens Pf0-1, pfs: P. fluorescens SBW25, pmy: P. mendocina ymp, ppf: P. putida F1, ppg: P. putida GB-1 ppu: P. putida KT2440, ppw: P. putida W619, psa: P. stutzeri A1501, psb: P. syringae pv. syringae B728a, psp: P. syringae pv. phaseolicola 1448A, and pst: P. syringae pv. tomato DC3000. (0.03 MB PDF) [file pcbi.1000868.s012.pdf]

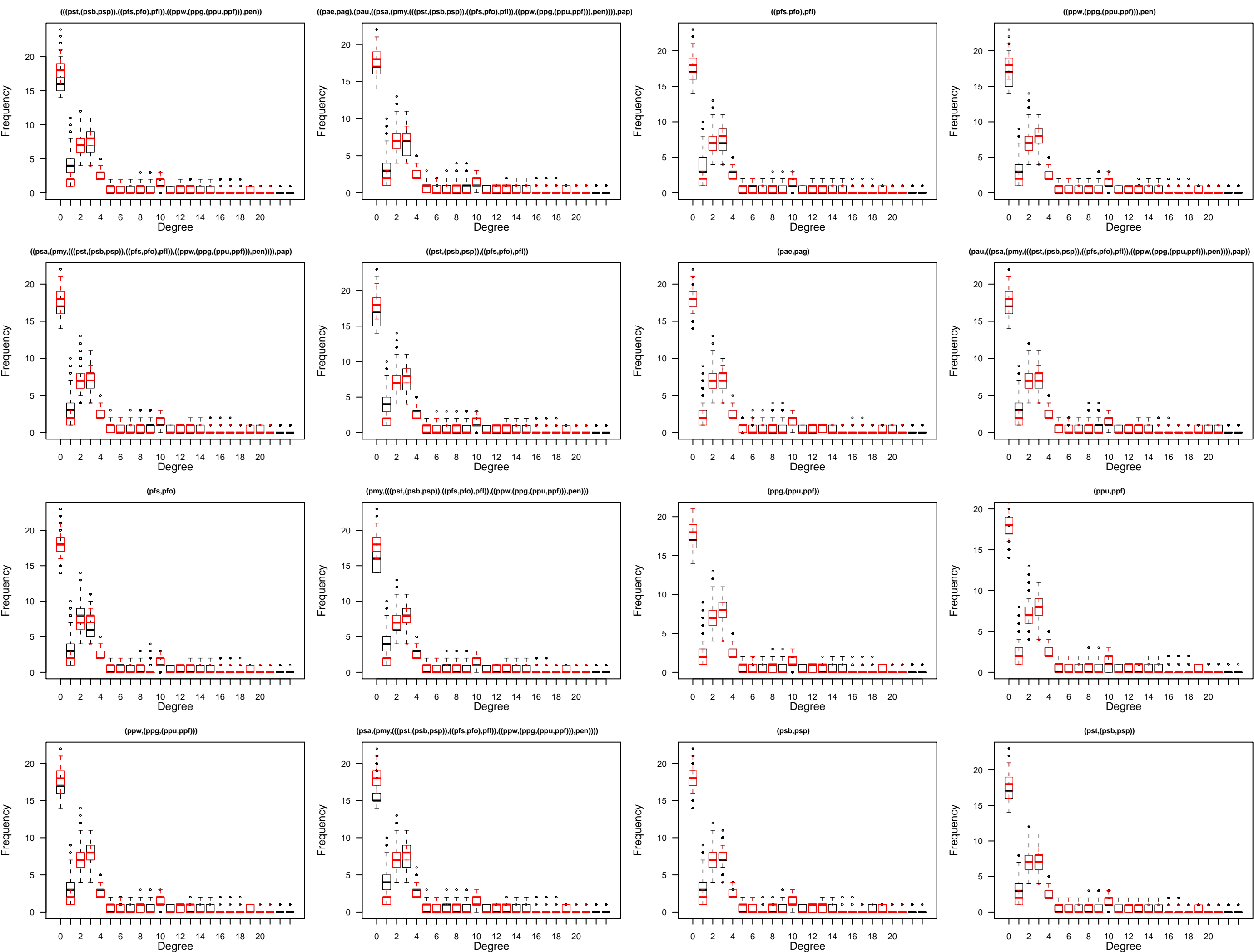

Supplement: Figure S13 — Degree distributions of nodes at the ancestral levels of the Pseudomonas phylogney shown in Figure 6A for the pyruvate metabolism map obtained using the Gibbs sampler. The actual degree distributions observed for the seventeen genome-sequenced Pseudomonas strains are shown in red. Strain abbreviations: pae: P. aeruginosa PAO1, pap: P. aeruginosa PA7, pau: P. aeruginosa PA14, pag: P. aeruginosa LESB58, pen: P. entomophila L48, pfl: P. fluorescens Pf-5, pfo: P. fluorescens Pf0-1, pfs: P. fluorescens SBW25, pmy: P. mendocina ymp, ppf: P. putida F1, ppg: P. putida GB-1 ppu: P. putida KT2440, ppw: P. putida W619, psa: P. stutzeri A1501, psb: P. syringae pv. syringae B728a, psp: P. syringae pv. phaseolicola 1448A, and pst: P. syringae pv. tomato DC3000. (0.08 MB PDF) [file pcbi.1000868.s013.pdf]
