# Supplementary material for: A Bayesian Approach to the Evolution of Metabolic Networks on a Phylogeny
Source: PLoS Comput Biol. 2010 Aug 5;6(8):e1000868. doi: 10.1371/journal.pcbi.1000868 (PMC2917375; doi:10.1371/journal.pcbi.1000868)
Supplement: Figure S6 — Number of insertion and deletion events for the alterable reactions, that is the reactions which were neither defined as core nor were defined as prohibited in the network obtained using the Gibbs sampler run under the hybrid model. The sampler was run for the six pathway maps used in this analysis over the phylogeny connecting the seventeen Pseudomonas strains shown in Figure 6A for 110,000 iterations with the first 10,000 iterations regarded as burn-in period. Samples were collected every 10th iteration. (0.02 MB PDF) [file pcbi.1000868.s006.pdf]

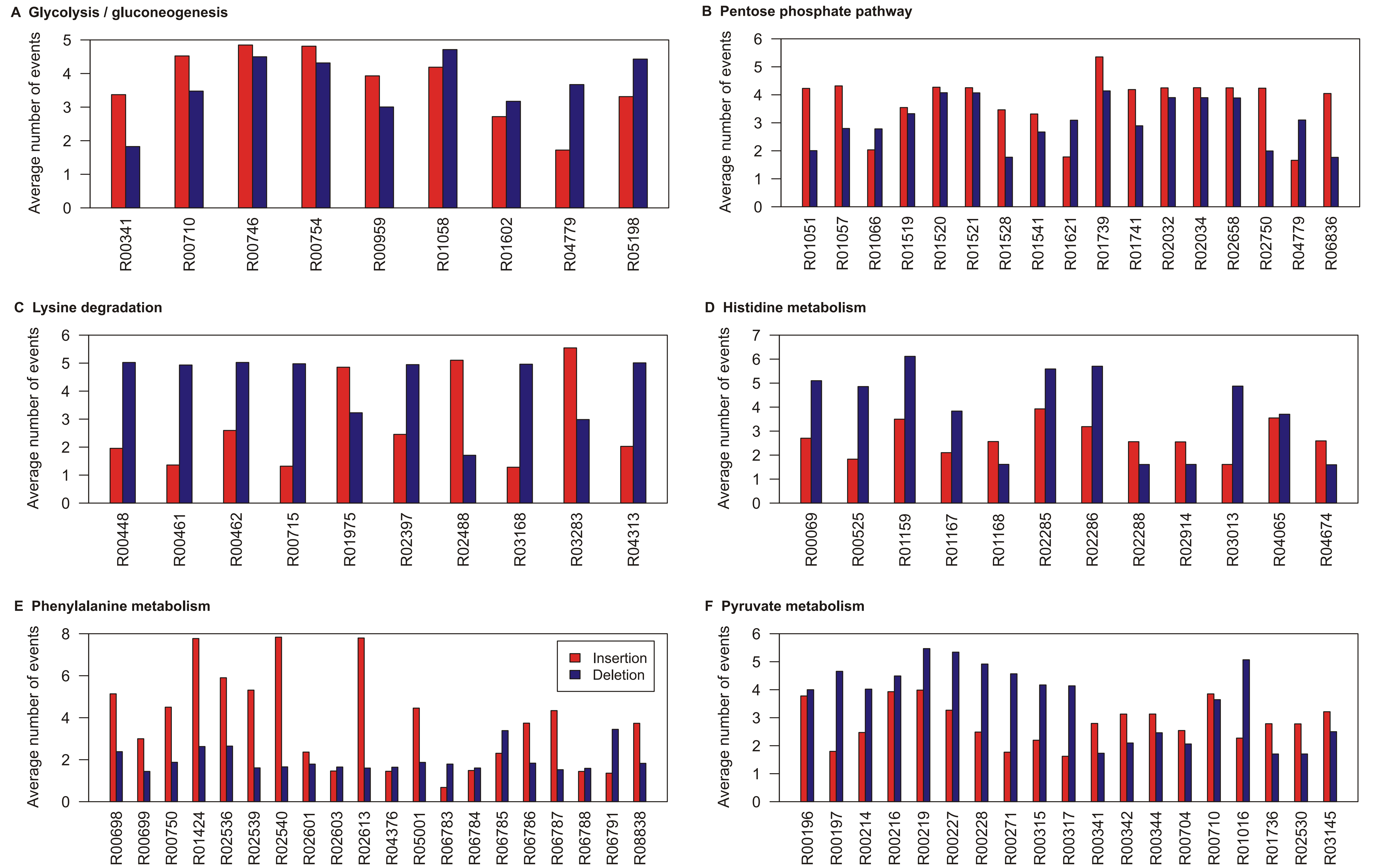
A Glycolysis / gluconeogenesis
B Pentose phosphate pathway
C Lysine degradation
D Histidine metabolism
E Phenylalanine metabolism
F Pyruvate metabolism
Average number of events
R00341
R00710
R00746
R00754
R00959
R01058
R01602
R04779
R05198
R01051
R01057
R01066
R01519
R01520
R01521
R01528
R01541
R01621
R01739
R01741
R02032
R02034
R02658
R02750
R04779
R06836
R00448
R00461
R00462
R00715
R01975
R02397
R02488
R03168
R03283
R04313
R00069
R00525
R01159
R01167
R01168
R02285
R02286
R02288
R02914
R03013
R04065
R04674
Insertion
Deletion
R00698
R00699
R00750
R01424
R02536
R02539
R02540
R02601
R02603
R02613
R04376
R05001
R06783
R06784
R06785
R06786
R06787
R06788
R06791
R08838
R00196
R00197
R00214
R00216
R00219
R00227
R00228
R00271
R00315
R00317
R00341
R00342
R00344
R00704
R00710
R01016
R01736
R02530
R03145
