# Supplementary material for: A Bayesian Approach to the Evolution of Metabolic Networks on a Phylogeny
Source: PLoS Comput Biol. 2010 Aug 5;6(8):e1000868. doi: 10.1371/journal.pcbi.1000868 (PMC2917375; doi:10.1371/journal.pcbi.1000868)
Supplement: Figure S7 — Number of insertion and deletion events at each branch of the phylogeny connecting the seventeen Pseudomonas strains shown in Figure 6A obtained using the Gibbs sampler run under the hybrid model. The sampler was run for 110,000 iterations with the first 10,000 iterations regarded as burn-in period. Samples were collected every 10th iteration. Strain abbreviations: pae: P. aeruginosa PAO1, pap: P. aeruginosa PA7, pau: P. aeruginosa PA14, pag: P. aeruginosa LESB58, pen: P. entomophila L48, pfl: P. fluorescens Pf-5, pfo: P. fluorescens Pf0-1, pfs: P. fluorescens SBW25, pmy: P. mendocina ymp, ppf: P. putida F1, ppg: P. putida GB-1 ppu: P. putida KT2440, ppw: P. putida W619, psa: P. stutzeri A1501, psb: P. syringae pv. syringae B728a, psp: P. syringae pv. phaseolicola 1448A, and pst: P. syringae pv. tomato DC3000. (0.02 MB PDF) [file pcbi.1000868.s007.pdf]

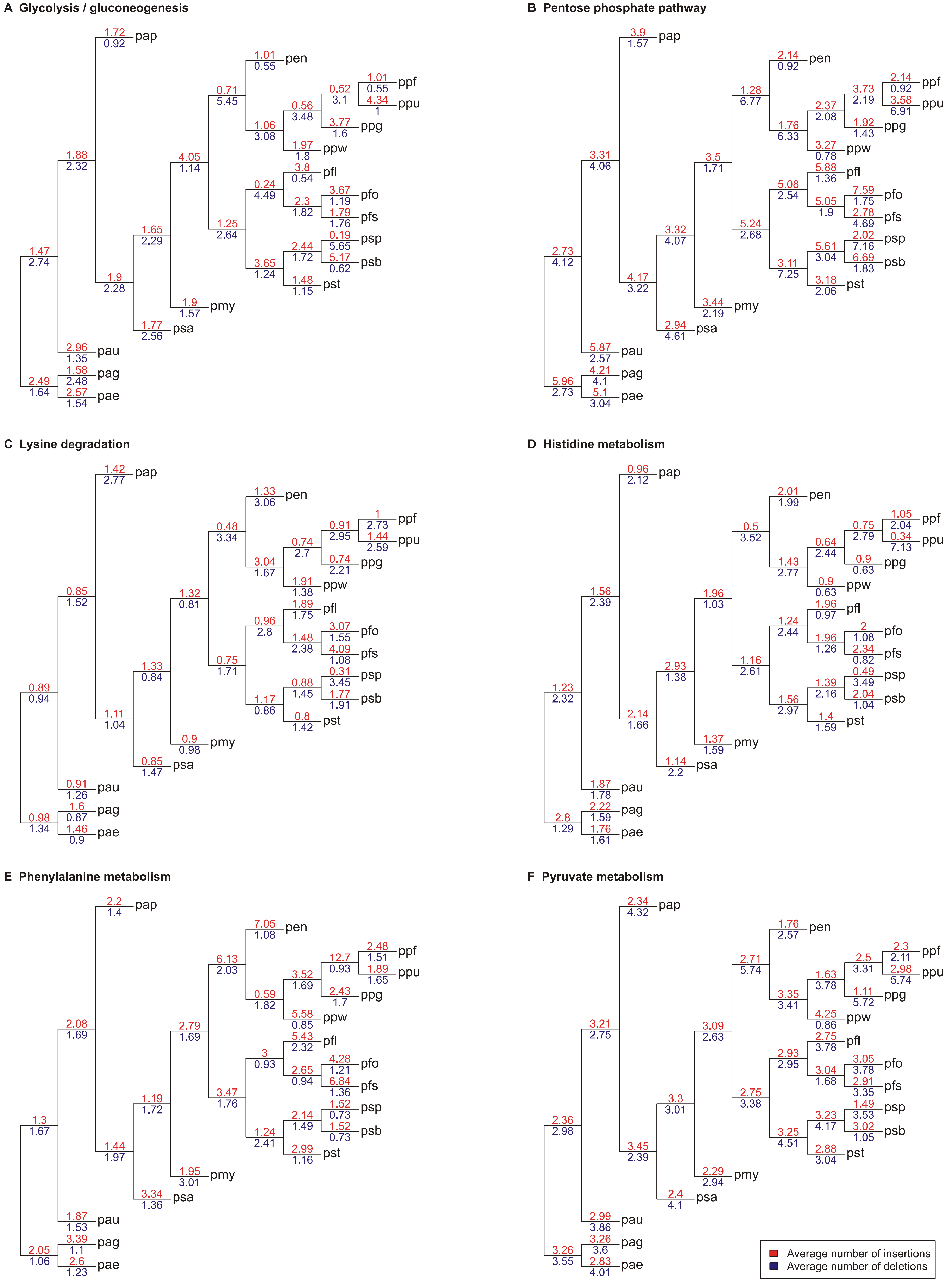
A Glycolysis / gluconeogenesis
B Pentose phosphate pathway
C Lysine degradation
D Histidine metabolism
E Phenylalanine metabolism
F Pyruvate metabolism
pap
pen
ppf
ppu
ppg
ppw
pfl
pfo
pfs
psp
psb
pst
pmy
psa
pau
pag
pae
Average number of insertions
Average number of deletions
