# Supplementary material for: A Bayesian Approach to the Evolution of Metabolic Networks on a Phylogeny
Source: PLoS Comput Biol. 2010 Aug 5;6(8):e1000868. doi: 10.1371/journal.pcbi.1000868 (PMC2917375; doi:10.1371/journal.pcbi.1000868)
Supplement: Table S1 — Pseudo-likelihood P*(H2|H1) conditioned on H for the toy networks shown in Figure 1 using different sub-network sizes. Also shown are the exact values calculated by full network exponentiation and by using the MCMC approach based on path sampling described in Mithani et al. [9] with 11,000 iterations including the first 1,000 iteration as burn-in period. (0.03 MB PDF) [file pcbi.1000868.s014.pdf]

Table S1

Parameters	$P^{\text{exact}}(H_2\|H_1)$	$P^{\text{MCMC}}(H_2\|H_1)$	Sub Network Size	$P^{*}(H_2\|H_1)$
$\lambda t = 0.1, \mu t = 0.2$	3.83215×10^{-3}	3.83215×10^{-3}	2	1.35067×10^{-4}
(Case: $\mu = 2\lambda$)			4	9.81626×10^{-4}
			6	2.53667×10^{-3}
			8	3.78735×10^{-3}
$\lambda t = 0.1, \mu t = 0.1$	2.40262×10^{-3}	2.40183×10^{-3}	2	7.23895×10^{-5}
(Case: $\mu = \lambda$)			4	5.62823×10^{-4}
			6	1.44553×10^{-3}
			8	2.66125×10^{-3}
$\lambda t = 0.1, \mu t = 0.05$	1.34591×10^{-3}	1.34526×10^{-3}	2	3.74695×10^{-5}
(Case: $\mu = \lambda/2$)			4	3.01569×10^{-4}
			6	7.71806×10^{-4}
			8	1.57826×10^{-3}
