# Supplementary material for: A Bayesian Approach to the Evolution of Metabolic Networks on a Phylogeny
Source: PLoS Comput Biol. 2010 Aug 5;6(8):e1000868. doi: 10.1371/journal.pcbi.1000868 (PMC2917375; doi:10.1371/journal.pcbi.1000868)
Supplement: Table S2 — Basic information of the metabolic networks for the seventeen genome-sequenced strains of Pseudomonas used in this study. A reversible reaction was represented by two hyperedges (one in either direction) in this study. The codes MAPxxxxx correspond to the respective KEGG pathway codes [22]. (0.04 MB PDF) [file pcbi.1000868.s015.pdf]

Table S2

Pathway Map	Organism	Reactions	Reversible	Hyperedges	Metabolites
Glycolysis / gluconeogenesis (MAP00010)	Reference Network	47	38	85	58
	P. aeruginosa LESB58	20	18	38	33
	P. aeruginosa PA14	23	21	44	37
	P. aeruginosa PA7	20	18	38	33
	P. aeruginosa PAO1	25	23	48	39
	P. entomophila L48	23	21	44	36
	P. fluorescens Pf0-1	26	24	50	38
	P. fluorescens Pf-5	26	24	50	40
	P. fluorescens SBW25	23	21	44	35
	P. mendocina ymp	22	20	42	35
	P. putida F1	24	22	46	38
	P. putida GB-1	21	19	40	34
	P. putida KT2440	25	23	48	39
	P. putida W619	22	20	42	35
	P. stutzeri A1501	25	23	48	38
	P. syringae pv. phaseolicola 1448A	25	23	48	38
	P. syringae pv. syringae B728a	25	23	48	38
	P. syringae pv. tomato DC3000	25	23	48	38
Pentose phosphate pathway (MAP00030)	Reference Network	42	40	82	57
	P. aeruginosa LESB58	21	20	41	37
	P. aeruginosa PA14	26	25	51	40
	P. aeruginosa PA7	25	24	49	40
	P. aeruginosa PAO1	26	25	51	40
	P. entomophila L48	22	21	43	36
	P. fluorescens Pf0-1	27	26	53	42
	P. fluorescens Pf-5	28	28	56	42
	P. fluorescens SBW25	22	21	43	38
	P. mendocina ymp	19	18	37	33
	P. putida F1	27	26	53	42
	P. putida GB-1	25	24	49	41
	P. putida KT2440	26	25	51	41
	P. putida W619	25	24	49	41
	P. stutzeri A1501	20	19	39	31
	P. syringae pv. phaseolicola 1448A	27	26	53	41
	P. syringae pv. syringae B728a	27	26	53	41
	P. syringae pv. tomato DC3000	27	26	53	41
Lysine degradation (MAP00310)	Reference Network	55	30	85	83
	P. aeruginosa LESB58	5	5	10	14
	P. aeruginosa PA14	7	6	13	19
	P. aeruginosa PA7	6	6	12	17
	P. aeruginosa PAO1	7	6	13	19
	P. entomophila L48	11	7	18	29
	P. fluorescens Pf0-1	8	6	14	23
	P. fluorescens Pf-5	9	7	16	25
	P. fluorescens SBW25	6	5	11	19
	P. mendocina ymp	9	7	16	25
	P. putida F1	8	7	15	21
	P. putida GB-1	6	6	12	17
	P. putida KT2440	7	6	13	19
	P. putida W619	7	6	13	19
	P. stutzeri A1501	7	6	13	19
	P. syringae pv. phaseolicola 1448A	7	4	11	22
	P. syringae pv. syringae B728a	6	5	11	19
	P. syringae pv. tomato DC3000	7	5	12	24

(continued)

Pathway Map	Organism	Reactions	Reversible	Hyperedges	Metabolites
Histidine metabolism	Reference Network	49	17	66	70
(MAP00340)	*P. aeruginosa* LESB58	14	11	25	30
	P. aeruginosa PA14	16	12	28	33
	P. aeruginosa PA7	14	11	25	30
	P. aeruginosa PAO1	16	11	27	35
	P. entomophila L48	18	12	30	38
	P. fluorescens Pf0-1	17	11	28	34
	P. fluorescens Pf-5	18	11	29	37
	P. fluorescens SBW25	15	11	26	32
	P. mendocina ymp	14	11	25	28
	P. putida F1	16	12	28	34
	P. putida GB-1	15	12	27	32
	P. putida KT2440	16	12	28	34
	P. putida W619	16	12	28	34
	P. stutzeri A1501	11	7	18	28
	P. syringae pv. phaseolicola 1448A	17	10	27	36
	P. syringae pv. syringae B728a	15	11	26	32
	P. syringae pv. tomato DC3000	15	11	26	32
Phenylalanine metabolism	Reference Network	59	21	80	73
(MAP00360)	*P. aeruginosa* LESB58	8	5	13	26
	P. aeruginosa PA14	8	5	13	26
	P. aeruginosa PA7	9	6	15	27
	P. aeruginosa PAO1	8	5	13	26
	P. entomophila L48	12	6	18	33
	P. fluorescens Pf0-1	8	4	12	28
	P. fluorescens Pf-5	10	5	15	31
	P. fluorescens SBW25	12	6	18	36
	P. mendocina ymp	5	3	8	17
	P. putida F1	13	6	19	39
	P. putida GB-1	11	6	17	32
	P. putida KT2440	11	5	16	31
	P. putida W619	19	7	26	46
	P. stutzeri A1501	6	4	10	19
	P. syringae pv. phaseolicola 1448A	9	5	14	29
	P. syringae pv. syringae B728a	9	5	14	26
	P. syringae pv. tomato DC3000	8	4	12	28
Pyruvate metabolism	Reference Network	71	50	121	76
(MAP00620)	*P. aeruginosa* LESB58	27	22	49	43
	P. aeruginosa PA14	29	24	53	44
	P. aeruginosa PA7	28	23	51	43
	P. aeruginosa PAO1	29	24	53	44
	P. entomophila L48	27	22	49	43
	P. fluorescens Pf0-1	26	21	47	40
	P. fluorescens Pf-5	27	20	47	43
	P. fluorescens SBW25	24	21	45	43
	P. mendocina ymp	28	22	50	46
	P. putida F1	29	23	52	45
	P. putida GB-1	25	21	46	43
	P. putida KT2440	24	20	44	41
	P. putida W619	27	23	50	44
	P. stutzeri A1501	28	23	51	45
	P. syringae pv. phaseolicola 1448A	27	22	49	43
	P. syringae pv. syringae B728a	27	23	50	45
	P. syringae pv. tomato DC3000	26	22	48	40
