# Supplementary material for: A Bayesian Approach to the Evolution of Metabolic Networks on a Phylogeny
Source: PLoS Comput Biol. 2010 Aug 5;6(8):e1000868. doi: 10.1371/journal.pcbi.1000868 (PMC2917375; doi:10.1371/journal.pcbi.1000868)
Supplement: Table S3 — Effective sample sizes (ESS) for the estimated parameters (δ: neighbor dependence probability, λ: insertion rate and μ: deletion rate) using the Gibbs sampler run under the hybrid model for the evolution of metabolic networks over the phylogeny connecting different Pseudomonas strains (Figure 6). Hyperedges that were common to all seventeen strains were regarded as core edges and hyperedges missing in all seventeen strains were regarded as prohibited edges. The values are averaged across three runs of 60,000 iterations for P. fluorescens and P. syringae phylogenies, and 110,000 iterations for the phylogeny connecting the seventeen Pseudomonas strains with the first 10,000 iterations regarded as burn-in period in each case. Samples were collected every 10th iteration. Strain abbreviations: pfl: Pseudomonas fluorescens Pf-5, pfo: Pseudomonas fluorescens Pf0-1, pfs: Pseudomonas fluorescens SBW25, psb: Pseudomonas syringae pv. syringae B728a, psp: Pseudomonas syringae pv. phaseolicola 1448A, and pst: Pseudomonas syringae pv. tomato DC3000. (0.03 MB PDF) [file pcbi.1000868.s016.pdf]

Table S3

Pathway Map	Phylogeny	ESS$_\delta$	ESS$_\lambda$	ESS$_\mu$
Glycolysis / Gluconeogenesis	((pfs,pfo),pfl)	2310	2310	2310
(MAP00010)	(pst,(psb,psp))	2310	2310	2310
	17 *Pseudomonas* strains	1626	1565	3099
Pentose phosphate pathway	((pfs,pfo),pfl)	757	539	401
(MAP00030)	(pst,(psb,psp))	2310	2310	1948
	17 *Pseudomonas* strains	2310	2310	1948
Lysine degradation	((pfs,pfo),pfl)	2310	2310	2310
(MAP00310)	(pst,(psb,psp))	2310	2310	2310
	17 *Pseudomonas* strains	1626	1565	3099
Histidine metabolism	((pfs,pfo),pfl)	2310	2310	2310
(MAP00340)	(pst,(psb,psp))	2310	2310	2310
	17 *Pseudomonas* strains	2310	2310	3897
Phenyalanine metabolism	((pfs,pfo),pfl)	2310	3897	2310
(MAP00360)	(pst,(psb,psp))	2310	1948	2310
	17 *Pseudomonas* strains	3128	3128	3099
Pyruvate metabolism	((pfs,pfo),pfl)	1224	825	956
(MAP00620)	(pst,(psb,psp))	2310	2310	3173
	17 *Pseudomonas* strains	1626	1565	3099
