# Supplementary material for: A Bayesian Approach to the Evolution of Metabolic Networks on a Phylogeny
Source: PLoS Comput Biol. 2010 Aug 5;6(8):e1000868. doi: 10.1371/journal.pcbi.1000868 (PMC2917375; doi:10.1371/journal.pcbi.1000868)
Supplement: Table S4 — Running time and the acceptance percentage of the Gibbs sampler for the estimation of evolution parameters (δ: neighbor dependence probability, λ: insertion rate and μ: deletion rate) run under the hybrid model for different metabolic networks over the phylogeny connecting different Pseudomonas strains (Figure 6). Hyperedges that were common to all seventeen strains were regarded as core edges and hyperedges missing in all seventeen strains were regarded as prohibited edges. The values are averaged across three runs of 60,000 iterations for P. fluorescens and P. syringae phylogenies, and 110,000 iterations for the phylogeny connecting the seventeen Pseudomonas strains with the first 10,000 iterations regarded as burn-in period in each case. Samples were collected every 10th iteration. Strain abbreviations: pfl: Pseudomonas fluorescens Pf-5, pfo: Pseudomonas fluorescens Pf0-1, pfs: Pseudomonas fluorescens SBW25, psb: Pseudomonas syringae pv. syringae B728a, psp: Pseudomonas syringae pv. phaseolicola 1448A, and pst: Pseudomonas syringae pv. tomato DC3000. (0.03 MB PDF) [file pcbi.1000868.s017.pdf]

Table S4

**Pathway Map**	**Phylogeny**	**Running Time** (hr)	**Acceptance Percentage**
Glycolysis / Gluconeogenesis	((pfs,pfo),pfl)	6.33 ± 0.55	14.11 ± 0.04
(MAP00010)	(pst,(psb,psp))	4.96 ± 0.18	33.28 ± 1.25
	17 *Pseudomonas* strains	44.41 ± 8.16	1.02 ± 0.06
Pentose phosphate pathway	((pfs,pfo),pfl)	12.37 ± 0.69	5.36 ± 0.04
(MAP00030)	(pst,(psb,psp))	10.16 ± 0.31	19.74 ± 0.34
	17 *Pseudomonas* strains	90.02 ± 8.25	1.01 ± 0.04
Lysine degradation	((pfs,pfo),pfl)	6.45 ± 0.99	27.26 ± 0.21
(MAP00310)	(pst,(psb,psp))	6.19 ± 0.88	23.65 ± 0.21
	17 *Pseudomonas* strains	38.47 ± 7.87	2.10 ± 0.72
Histidine metabolism	((pfs,pfo),pfl)	5.52 ± 0.52	22.23 ± 0.31
(MAP00340)	(pst,(psb,psp))	5.65 ± 0.36	22.91 ± 0.30
	17 *Pseudomonas* strains	29.54 ± 7.21	1.08 ± 0.09
Phenyalanine metabolism	((pfs,pfo),pfl)	9.39 ± 0.84	15.47 ± 0.21
(MAP00360)	(pst,(psb,psp))	9.20 ± 0.85	14.16 ± 0.09
	17 *Pseudomonas* strains	52.49 ± 5.04	1.59 ± 0.07
Pyruvate metabolism	((pfs,pfo),pfl)	23.76 ± 1.92	8.66 ± 0.06
(MAP00620)	(pst,(psb,psp))	24.20 ± 2.05	15.13 ± 0.24
	17 *Pseudomonas* strains	98.01 ± 9.10	1.29 ± 0.03
