# Supplementary material for: A Bayesian Approach to the Evolution of Metabolic Networks on a Phylogeny
Source: PLoS Comput Biol. 2010 Aug 5;6(8):e1000868. doi: 10.1371/journal.pcbi.1000868 (PMC2917375; doi:10.1371/journal.pcbi.1000868)
Supplement: Figure S8 — Degree distributions of nodes at the ancestral levels of the Pseudomonas phylogney shown in Figure 6A for the glycolysis/gluconeogenesis map obtained using the Gibbs sampler. The actual degree distributions observed for the seventeen genome-sequenced Pseudomonas strains are shown in red. Strain abbreviations: pae: P. aeruginosa PAO1, pap: P. aeruginosa PA7, pau: P. aeruginosa PA14, pag: P. aeruginosa LESB58, pen: P. entomophila L48, pfl: P. fluorescens Pf-5, pfo: P. fluorescens Pf0-1, pfs: P. fluorescens SBW25, pmy: P. mendocina ymp, ppf: P. putida F1, ppg: P. putida GB-1 ppu: P. putida KT2440, ppw: P. putida W619, psa: P. stutzeri A1501, psb: P. syringae pv. syringae B728a, psp: P. syringae pv. phaseolicola 1448A, and pst: P. syringae pv. tomato DC3000. (0.04 MB PDF) [file pcbi.1000868.s008.pdf]

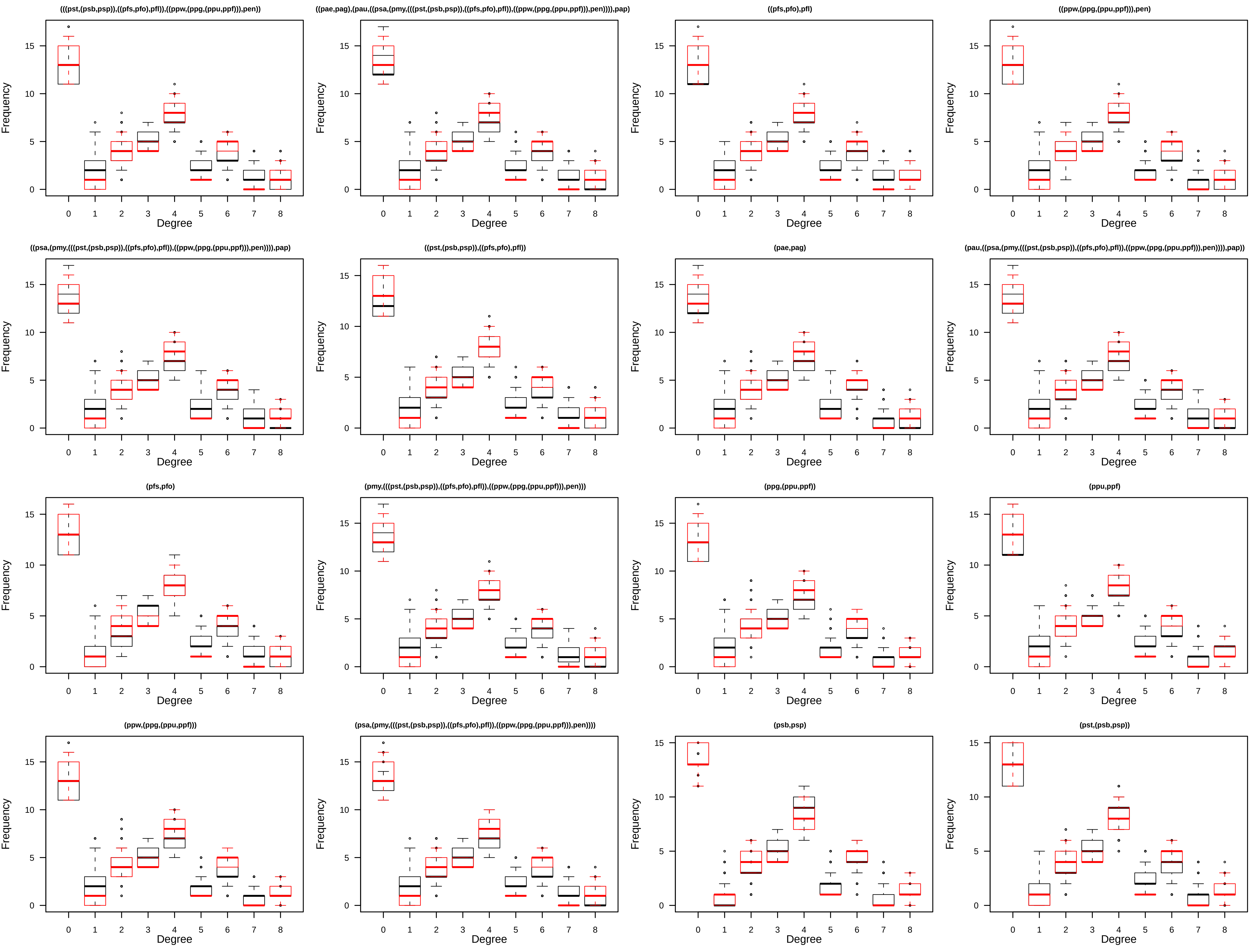
(((pst,(psb,psp)),((pfs,pfo),pfl)),((ppw,(ppg,(ppu,ppf))),pen))
((pae,pag),(pau,((psa,(pmy,(((pst,(psb,psp)),((pfs,pfo),pfl)),((ppw,(ppg,(ppu,ppf))),pen)))),pap))
((pfs,pfo),pfl)
((ppw,(ppg,(ppu,ppf))),pen)
((psa,(pmy,(((pst,(psb,psp)),((pfs,pfo),pfl)),((ppw,(ppg,(ppu,ppf))),pen)))),pap)
((pst,(psb,psp)),((pfs,pfo),pfl))
(pae,pag)
(pau,((psa,(pmy,(((pst,(psb,psp)),((pfs,pfo),pfl)),((ppw,(ppg,(ppu,ppf))),pen)))),pap))
(pfs,pfo)
(pmy,(((pst,(psb,psp)),((pfs,pfo),pfl)),((ppw,(ppg,(ppu,ppf))),pen)))
(ppg,(ppu,ppf))
(ppu,ppf)
(ppw,(ppg,(ppu,ppf)))
(psa,(pmy,(((pst,(psb,psp)),((pfs,pfo),pfl)),((ppw,(ppg,(ppu,ppf))),pen))))
(psb,psp)
(pst,(psb,psp))
Frequency
Degree
0 1 2 3 4 5 6 7 8
0 5 10 15
