# Supplementary material for: A Bayesian Approach to the Evolution of Metabolic Networks on a Phylogeny
Source: PLoS Comput Biol. 2010 Aug 5;6(8):e1000868. doi: 10.1371/journal.pcbi.1000868 (PMC2917375; doi:10.1371/journal.pcbi.1000868)
Supplement: Figure S11 — Degree distributions of nodes at the ancestral levels of the Pseudomonas phylogney shown in Figure 6A for the histidine metabolism map obtained using the Gibbs sampler. The actual degree distributions observed for the seventeen genome-sequenced Pseudomonas strains are shown in red. Strain abbreviations: pae: P. aeruginosa PAO1, pap: P. aeruginosa PA7, pau: P. aeruginosa PA14, pag: P. aeruginosa LESB58, pen: P. entomophila L48, pfl: P. fluorescens Pf-5, pfo: P. fluorescens Pf0-1, pfs: P. fluorescens SBW25, pmy: P. mendocina ymp, ppf: P. putida F1, ppg: P. putida GB-1 ppu: P. putida KT2440, ppw: P. putida W619, psa: P. stutzeri A1501, psb: P. syringae pv. syringae B728a, psp: P. syringae pv. phaseolicola 1448A, and pst: P. syringae pv. tomato DC3000. (0.04 MB PDF) [file pcbi.1000868.s011.pdf]

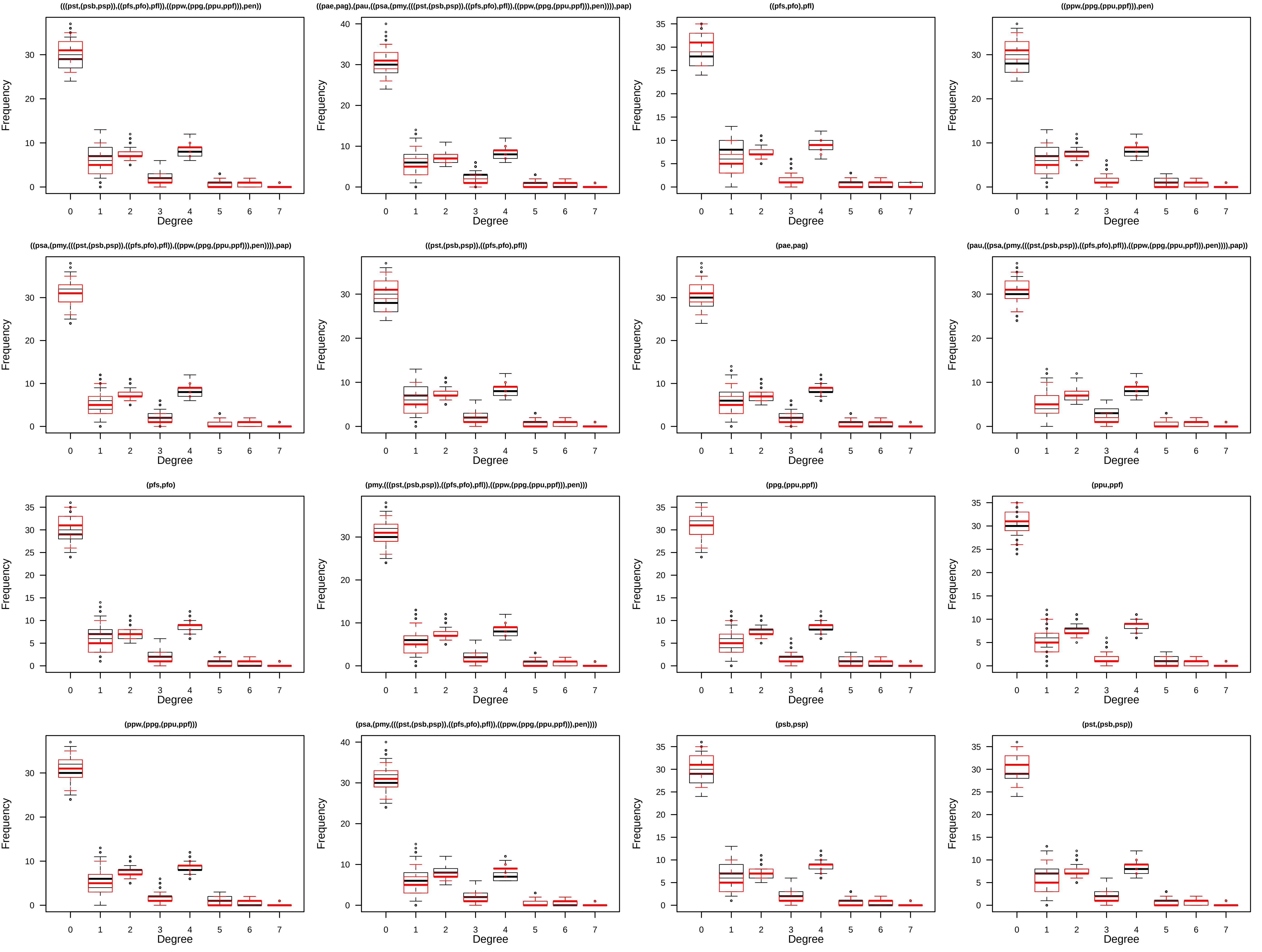
(((pst,(psb,psp)),((pfs,pfo),pfl)),((ppw,(ppg,(ppu,ppf))),pen))
((pae,pag),(pau,((psa,(pmy,(((pst,(psb,psp)),((pfs,pfo),pfl)),((ppw,(ppg,(ppu,ppf))),pen)))),pap)))
((pfs,pfo),pfl)
((ppw,(ppg,(ppu,ppf))),pen)
((psa,(pmy,(((pst,(psb,psp)),((pfs,pfo),pfl)),((ppw,(ppg,(ppu,ppf))),pen)))),pap)
((pst,(psb,psp)),((pfs,pfo),pfl))
(pae,pag)
(pau,((psa,(pmy,(((pst,(psb,psp)),((pfs,pfo),pfl)),((ppw,(ppg,(ppu,ppf))),pen)))),pap))
(pfs,pfo)
(pmy,(((pst,(psb,psp)),((pfs,pfo),pfl)),((ppw,(ppg,(ppu,ppf))),pen)))
(ppg,(ppu,ppf))
(ppu,ppf)
(ppw,(ppg,(ppu,ppf)))
(psa,(pmy,(((pst,(psb,psp)),((pfs,pfo),pfl)),((ppw,(ppg,(ppu,ppf))),pen))))
(psb,psp)
(pst,(psb,psp))
Frequency
Degree
