# Supplementary material for: A Bayesian Approach to the Evolution of Metabolic Networks on a Phylogeny
Source: PLoS Comput Biol. 2010 Aug 5;6(8):e1000868. doi: 10.1371/journal.pcbi.1000868 (PMC2917375; doi:10.1371/journal.pcbi.1000868)

Text S1

Testing of Gibbs sampler for parameter estimation

To test the Gibbs sampler for estimating the parameters of evolution described in Section 'Estimation of parameters', we simulated the network evolution on the phylogeny shown in Figure 1 of the paper and re-estimated the parameters from the simulated data. At each step of the simulation, networks at the internal nodes were sampled using the procedure described in Section 'Sampling internal nodes' with rate parameters $(\lambda, \mu) = (0.06, 0.03)$ and the neighbor-dependence probability $\delta = 1$. A total of 25,000 iterations were used as burn-in period and a further 50,000 iterations were run to obtain networks from the stationary distribution. Internal networks were sampled at every 10th iterations and the Gibbs sampler was used to estimate the parameters. We discuss the results for rate parameters here for example purposes. As the samples were taken from the stationary distribution, it was expected to get the ratio of the rate parameters back instead of the actual parameter values since the behavior of the system in the equilibrium condition is driven by the ratio (λ/μ) and not the actual parameters. The posterior mean of the ratio of the estimated rate parameters was calculated as 1.83 compared to the true ratio of 2.0 with posterior variance equaling to 0.1088. The density plot of the ratio (λ/μ) of estimated parameters for 5000 samples is shown below.

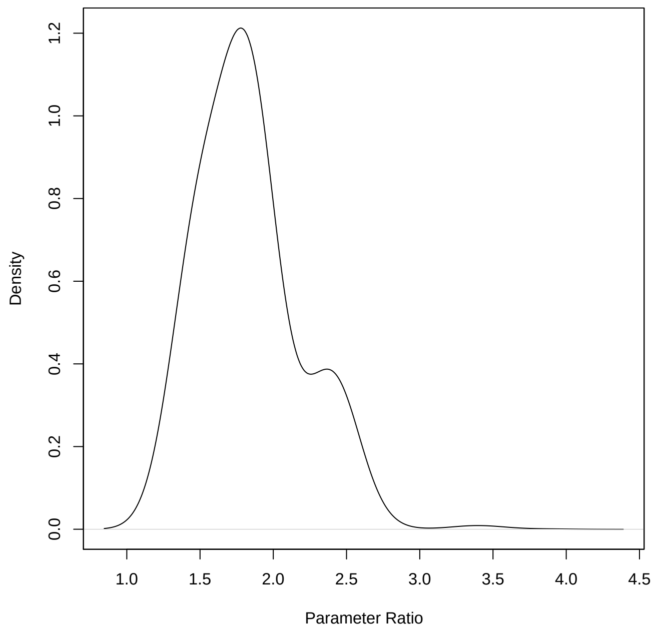

Supplement: Text S1 — Testing of the Gibbs sampler for parameter estimation. (0.04 MB PDF) [file pcbi.1000868.s018.pdf]
